# One-year trajectories of physical and mental health-related quality of life, fatigue and dyspnoea in COVID-19 survivors

**DOI:** 10.1007/s11136-024-03812-y

**Published:** 2024-10-19

**Authors:** Gerko Schaap, John F. Davelaar, Peter M. ten Klooster, Carine J. M. Doggen, Job van der Palen, Christina Bode, Harald E. Vonkeman

**Affiliations:** 1 Section of Psychology, Health & Technology, University of Twente, Enschede, The Netherlands; 2 Department of Rheumatology and Clinical Immunology, Medisch Spectrum Twente, Enschede, The Netherlands; 3 Section of Health Technology and Services Research, Technical Medical Centre, University of Twente, Enschede, The Netherlands; 4 Clinical Research Centre, Rijnstate Hospital, Arnhem, The Netherlands; 5 Section of Cognition, Data and Education, University of Twente, Enschede, The Netherlands; 6 Department of Epidemiology, Medisch Spectrum Twente, Enschede, The Netherlands

**Keywords:** COVID-19, Post-COVID-19 syndrome, Quality of life, Fatigue, Dyspnoea, Latent class growth mixture modelling

## Abstract

**Purpose:**

A substantial number of people experience a persisting impact on health-related quality of life (HRQoL) after COVID-19. The current study aims to identify different trajectories of physical and mental HRQoL, fatigue severity, and dyspnoea severity following hospitalisation with COVID-19, and associated factors of these trajectories.

**Methods:**

500 patients with COVID-19 were followed for one year in a longitudinal cohort study. Self-reported outcomes were measured at 3, 6, 9, and 12 months after hospitalisation. Distinct trajectories were characterised using Growth Mixture Modelling. Sociodemographic and clinical correlates of trajectories were investigated using multivariable (multinomial) logistic regression analyses.

**Results:**

Three trajectories (‘stable high’ (16%), ‘improving’ (40%), and ‘stable low’ (44%)) were found for physical HRQoL, and four (‘stable high’ (43%), ‘improving’ (14%), ‘middle declining’ (17%), and ‘low’ (26%)) for mental HRQoL. Older age, overweight and obesity, lower education, and comorbidities were associated with ‘low’ physical HRQoL. Younger age was associated with ‘low’ mental HRQoL. Four fatigue trajectories (‘no fatigue’ (15%), ‘improving’ (40%), ‘low-severe’ (27%), and ‘high-severe’ (18%)) were found. Participants either experienced almost never (‘no dyspnoea’, 75%) or almost always (‘severe’, 25%) dyspnoea. High co-occurrences between low HRQoL and severe fatigue and dyspnoea symptom trajectories were found.

**Conclusion:**

A substantial number of COVID-19 survivors continue to struggle with reduced HRQoL over time. However, large variations in these physical and mental HRQoL trajectories exist, and trajectories are associated with persisting COVID-19-related symptoms or pre-hospitalised health status. Regular measurement of HRQoL and post-COVID symptoms may help identify those that may benefit from timely interventions.

**Supplementary Information:**

The online version contains supplementary material available at 10.1007/s11136-024-03812-y.

## Introduction

As of February 2024, over 774 million people worldwide have been infected with SARS-CoV-2 at least once, and over seven million deaths have been attributed to COVID-19 [1]. Over the past three years, increasing attention has been given to the long-term impact of COVID-19 in patients, including post-COVID-19 syndrome – colloquially known as Long COVID. While the majority of COVID-19 patients recover well, a substantial number of people experience persisting symptoms for longer than 12 weeks since the initial infection, which cannot be attributed to alternative diagnoses [2]. The most frequently reported post-COVID-19 symptoms include fatigue and post-exertional malaise, respiratory complaints such as dyspnoea, pain, cognitive dysfunction – including attention and memory problems, and brain fog – and psychological symptoms such as anxiety and depression [3–5]. Accordingly, the health-related quality of life of COVID-19 patients – and especially post-COVID-19 patients – is often reduced.

Health-related quality of life (HRQoL) is a complex and multifaceted concept related to subjective experiences of physical, mental, and social well-being and functioning [6]. Previous studies have reported significantly reduced HRQoL in COVID-19 survivors, especially those with persisting symptoms [7–11]. Older age, female sex, higher body mass index (BMI), persisting fatigue and having comorbidities were identified as risk factors or associated factors in COVID-19 sequelae, including reduced HRQoL [10–13]. These studies are based mostly on cross-sectional measurements or singular follow-up measurements designs, and do not account for heterogeneity in how HRQoL develops over time. As COVID-19 recovery trajectories differ between patients, different trajectories in HRQoL are to be expected as well. For example, certain groups of patients may be characterised by a speedy recovery to (almost) normal HRQoL, while other groups recover slowly or not at all.

Moreover, many studies did not differentiate between physical and mental HRQoL. However, physical and mental health complaints may affect HRQoL aspects differently and require different interventions. Moreover, good physical HRQoL does not necessarily correlate with good mental HRQoL [13–15]. For example, people can struggle with somatic complaints, such as mobility restrictions, but function well mentally, and vice versa.

To address these gaps, the current study aims to identify different trajectories in physical and mental HRQoL following hospitalisation with COVID-19, and to identify associated factors of these trajectories. Additionally, heterogeneity in trajectories of fatigue and dyspnoea after COVID-19 have not yet been studied. These specific symptoms are frequently reported and can severely influence HRQoL. Insights into trajectories and associated factors can help support choices for intervention in COVID-19 survivors, such as the timing, target mechanisms, and target groups.

## Methods

### Study design and participants

This study was a retrospective analysis of an ongoing longitudinal cohort study consisting of self-report questionnaires in COVID-19 patients admitted to two hospitals in Twente, the Netherlands, starting after their discharge from the hospital (Clinical Trials: NCT05813574). Enrolment began on July 28, 2020. The study was approved by the Medisch Spectrum Twente Institutional Review Board (K20-30).

All patients hospitalised with a confirmed SARS-CoV-2 infection were asked to participate in this study following hospital discharge. No records were kept of how many patients declined participation and for which reasons. As non-respondents did not provide informed consent for accessing their health records, no comparisons of characteristics between respondents and non-respondents were possible. Inclusion criteria of the cohort study were: a) hospital admission with polymerase chain reaction confirmed SARS-CoV-2 infection; b) ≥ 18 years of age; c) proficiency in Dutch; d) providing informed consent. Additionally, for the current analysis participants were included if they responded to the HRQoL outcome measures of at least 3 out of 4 included measurement timepoints. Half of the participants (47%, *n* = 235) filled out all measurements and 53% (265 participants) filled out 3 out of 4 measurements. Of the excluded participants, 99 participants had 1 and 95 had 2 observations.

The current analysis included observations at 3, 6, 9, and 12 months after hospital discharge. Measurement points were estimated within ± 45 days windows of when the participant was required to answer the questionnaires. The questionnaires were administered in Dutch, either online using Qualtrics or on paper via postal mail, depending on the participant’s preferences. While baseline (discharge) questionnaires were administered, these were either not returned or returned > 45 days (around 2 months) after discharge, most likely not accurately capturing observations of the outcomes at baseline. Missing data of baseline were 75%. Hence, these measurements were excluded from this analysis. Questionnaires were completed on average at 93 (*SD* = 21.8), 184 (*SD* = 17.7), 272 (*SD* = 15.5), and 367 (*SD* = 14.7) days after hospital discharge for the 3, 6, 9, and 12 months measurements respectively.

The Guidelines for Reporting Latent Trajectory Studies (GRoLTS) Checklist [16] and STROBE statement [17] have been followed in reporting the current study (supplementary material).

### Measures

HRQoL was measured using the Dutch Short Form Health Survey 36 (SF-36) [18]. The SF-36 assesses HRQoL over eight different domains: physical functioning, physical role limitations, bodily pain, general health perceptions, vitality, social functioning, emotional role functioning and mental health. The domains were summarised in higher-order physical and mental HRQoL indices: the Physical Component Summary (PCS) and Mental Component Summary (MCS) scores. These scores were standardised (mean score = 50 and standard deviation = 10) using normative data from the United States general population as per standard instructions [19]. Higher scores indicate better HRQoL.

Fatigue severity was assessed with the Short Fatigue Questionnaire (SFQ) [20, 21]. Possible ranges are from 4 to 28, with higher scores indicating higher severity. A cut-off score of 18 indicates clinically severe fatigue [20]. Dyspnoea was assessed using the modified Medical Research Council (mMRC) dyspnoea scale [22]. The mMRC asks participants to grade the effect of dyspnoea on their daily life activities on a scale from 0 (no burden) to 4 (worst burden) [23]. A score ≥ 2 indicates clinically severe burden.

Sociodemographic and clinical characteristics were self-reported at baseline. Comorbidities were assessed by asking whether a condition (e.g. cancer, lung condition, depression, ‘other’) was present or not. These conditions were recoded using ICD-11 classifications. BMI was calculated as kilograms/(metres²) and categorised according to WHO guidelines to account for outliers. Highest completed education was categorised as low (none, only primary or lower secondary school or lower vocational school), medium (higher secondary school or medium vocational school) or high (higher vocational school or university grade).

### Data analysis

Growth Mixture Modelling (GMM) was applied to investigate unobserved heterogeneity in development trajectories. GMM is a person-centred approach which aims to classify individuals into distinct categories (classes) based on their own patterns of an outcome measure over time [24]. The optimal number of classes was determined via statistical criteria and clinical interpretability. The lowest Bayesian Information Criterion (BIC) value was primarily used to identify the best fit, but sample size-adjusted BIC (saBIC) and Akaike Information Criterion (AIC) indices were also considered. The accuracy of the classification was evaluated using the entropy index, with values closer to 1 representing a higher adequacy. Promising trajectory solutions were plotted and analysed visually for clinical meaningfulness. Additional information, detailed descriptions and fit indices of the model selection are provided in the supplementary material.

Quadratic model structures with random intercepts and slopes and unstructured variance-covariance matrix were favoured for physical and mental HRQoL and fatigue. Explorative investigations identified that GMM for dyspnoea was not feasible, as little variation over time could be identified (supplementary material). Thus, participants were classified into a ‘severe’ trajectory when they indicated severe scores for the majority of observations and as ‘no dyspnoea’ otherwise.

Associations of sociodemographic and clinical characteristics with trajectories were investigated using the standard three-step method [16]. After the final model was run without covariates (step 1), most likely class membership was saved and merged with the original dataset (step 2). Next, characteristics were analysed using multivariable multinomial logistic regression (step 3). The trajectories indicative of the best health over time were set as reference categories. For dyspnoea, multivariable binary logistic regression was used. Relative risk ratios (RRR) for multinomial regression and odds ratios for binary regression are presented with 95% confidence intervals. Statistical significance was set as *p* < 0.05.

To explore associations between the four HRQoL and symptom severity trajectories, frequencies of the combinations of trajectories were summarised in a table. Combinations are presented when they occur in at least ten participants.

Missing data were assumed to be missing at random (MAR) and no imputation methods were used. The HRQoL, fatigue and dyspnoea measures had missing values around 25% for 3 months, 11% for 6 months, 10% for 9 months, and 12% for 12 months respectively, which were handled using mixed modelling (i.e. full information maximum likelihood estimations) for GMM or listwise deletion for dyspnoea. Baseline characteristics were handled using listwise deletion as only a small proportion (around 2.5%) of the characteristics were missing. Analyses were performed in R v4.2.2 [25]. GMM were run using the function *hlme* from the *lcmm* v2.0.2 package [26]. Descriptive statistics were analysed with the package *gtsummary* v1.7.2 [27]. The function *mulinom* from the package *nnet* v7.3-18 was used for multinomial logistic regression analyses [28].

## Results

### Sample characteristics

Participants’ characteristics are presented in Table 1. Of the 500 included participants, ages ranged between 32 and 89 (Mean = 63.9, SD = 10.9), and two third (63%) were men. On average, participants had above normal weight (80%), and about half (47%) had a medium level education. The majority (77%) had at least one comorbidity at the time of their first questionnaire. No differences between the two hospitals or between the sample and participants who dropped out (*N* = 194) were found (Table S5).

**Table 1:** Sample characteristics of 500 patients hospitalised with COVID-19

	Mean/*n*	SD/%
Age, Mean (*SD*)	63.9	10.9
Sex, *n* (%)		
Men	315	63
Women	185	37
BMI classification, *n* (%)		
Normal weight (18.5–25)	99	20
Overweight (25–30)	239	49
Obese (> 30)	151	31
Education, *n* (%)		
Low	114	23
Medium	232	47
High	145	30
Number of comorbidities, *n* (%)		
None	112	23
1	209	42
2	97	20
≥3	74	15
Comorbidities, *n* (%)		
Cancer	23	5
Cardiovascular	205	42
Digestive	44	9
Endocrine	83	17
Genitourinary	17	4
Musculoskeletal	81	17
Neurological	25	5
Psychiatric	18	4
Respiratory	129	26
Other ^a^	30	6

Notes. ^a^ Blood disorders (3%), skin diseases (1%), allergic disorders (1%), and other conditions (all < 0.5%)

Distributions of the outcome variables in the overall sample, for each timepoint, and per trajectory are reported in supplementary Table S6. See also the supplementary material for detailed information of the trajectories, i.e. the individual trajectories for each outcome (Fig. S14-16) and detailed information on characteristics of identified classes (Tables S7 and S8).

### Physical HRQoL trajectories

**Fig. 1:**
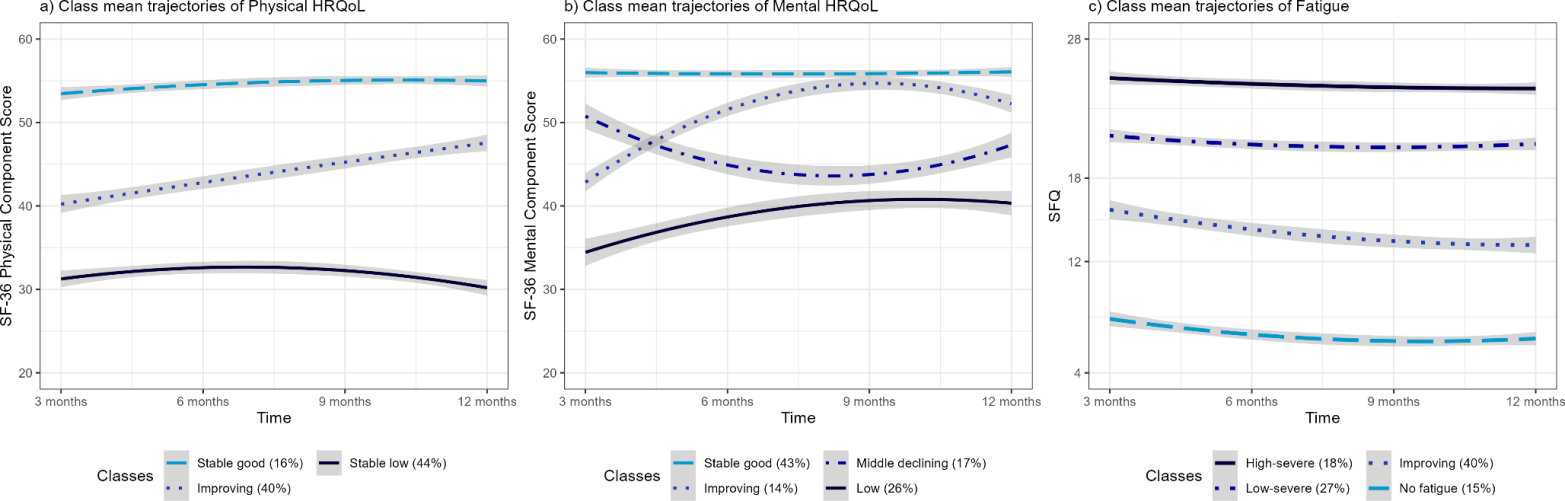
Class trajectories of health-related quality of life and fatigue of participants over one year *Notes*. Trajectories on a class level with a 95% confidence interval (grey) for respectively physical (**a**) and mental (**b**) health-related quality of life (HRQoL) as measured with the summary indices of the Short Form Health Survey 36 (*SF-36*; possible range 0–100) and fatigue (**c**) as measured with the Short Fatigue Questionnaire (SFQ)

Three main trajectories were identified for physical HRQoL (PCS; Fig. 1a). A small number of participants (16%, *n* = 79) seemed to recover well after COVID-19 and are characterised by above-average (‘stable good’) physical quality of life. Secondly, one class (40%, *n* = 201) was labelled ‘improving’ as it showed a below-average physical HRQoL three months after discharge that improved slightly over time to just below the norm score (50) of PCS for general populations [18]. Lastly, 44% (*n* = 220) of participants experienced considerable reduced physical HRQoL consistently after hospital discharge, labelled as ‘stable low’.

Multivariable logistic regression analysis (Table 2) showed that, compared to being in the ‘stable good’ trajectory, overweight and obesity, having a low or medium education, and having at least one comorbidity were associated with being classified in either the ‘improving’ or ‘stable low’ trajectories. Additionally, older age was associated with the ‘stable low’ trajectory.

### Mental HRQoL trajectories

For mental HRQoL (MCS), four main trajectories were found (Fig. 1b). The ‘stable good’ trajectory (43%, *n* = 213) showed a consistent, good mental HRQoL, while the ‘low’ trajectory (26%, *n* = 130) showed mental HRQoL to be far below the norm with limited improvement. Additionally, one small group (14%, *n* = 73), labelled ‘improving’, showed a development from somewhat below to just above the norm score, with a slight decrease after 9 months. Finally, one trajectory (17%, *n* = 84) was labelled as ‘middle declining’, as it started out around the norm score, but on average seemed to slightly decline over time, with after a low point at 9 months increasing again to just below the norm score.

There were no significant associations of characteristics with belonging to the ‘improving trajectory’ (Table 2). Having a low or medium education and having at least one comorbidity were associated with belonging to the ‘middle declining’ trajectory. Younger age was associated with being in the ‘low’ trajectory.

**Table 2:** Multivariable multinomial logistic regression models of associations of sociodemographic and clinical characteristics with health-related quality of life trajectory membership

	Physical HRQoL		Mental HRQoL		
	Improving trajectory	Stable low trajectory	Improving trajectory	Middle declining trajectory	Low trajectory
	RRR [95%CI]		RRR [95%CI]		
Age in years	0.99 [0.97; 1.02]	1.04 [1.01; 1.07]**	1.00 [0.97; 1.03]	0.98 [0.96; 1.01]	0.97 [0.95; 0.99]*
Sex (ref. = male)	1.22 [0.67; 2.23]	1.83 [0.99; 3.41]	1.27 [0.73; 2.23]	0.71 [0.41; 1.25]	1.11 [0.69; 1.77]
BMI (ref. = normal weight)					
Overweight	2.10 [1.10; 4.01]*	2.17 [1.09; 4.32]*	1.51 [0.72; 3.18]	1.01 [0.50; 2.03]	1.21 [0.62; 2.12]
Obese	2.45 [1.05; 5.70]*	6.14 [2.60; 14.54]***	1.48 [0.64; 3.40]	1.38 [0.65; 2.92]	1.47 [0.75; 2.88]
Education (ref. = low/medium)	0.54 [0.31; 0.95]*	0.38 [0.21; 0.70]**	0.95 [0.52; 1.72]	0.52 [0.28; 0.97]*	0.77 [0.47; 1.28]
Comorbidity (ref. = none)	2.01 [1.13; 3.55]*	4.76 [2.46; 9.22]***	1.30 [0.67; 2.52]	2.60 [1.25; 5.39]**	1.73 [0.99; 3.02]

*Notes*. ‘Stable good’ trajectories classes for both physical and mental health-related quality of life (HRQoL) were set as reference outcome categories. * *p* < 0.05; ** *p* < 0.01; *** *p* < 0.001. BMI = body mass index; CI = confidence intervals; HRQoL = health-related quality of life; ref. = reference category; RRR = relative risk ratio

Overall, fewer sociodemographic and clinical characteristics were associated with mental HRQoL development compared to physical HRQoL development.

### Fatigue trajectories

Four main trajectories were found for fatigue (SFQ; Fig. 1c). A small group of participants (15%, *n* = 77) consistently experienced no or very mild fatigue, labelled as the ‘no fatigue’ trajectory. Additionally, 40% (*n* = 199) showed moderate levels of fatigue with slight but stable improvement over time (‘improving’). Two groups identified participants with stable, severe fatigue. The ‘low-severe’ trajectory (27%, *n* = 133) showed severe fatigue symptoms typical for many chronic conditions. The ‘high-severe’ trajectory (18%, *n* = 91) consistently showed scores far above the cutoff (18), similar to patients diagnosed with chronic fatigue syndrome [20].

Overweight and obesity were associated with being in the ‘improving’ trajectory (Table 3). Obesity, having a low or medium education, and having at least one comorbidity were associated with being in the ‘low-severe’ trajectory. Female sex, obesity, and having at least one comorbidity were associated with being in the ‘high-severe’ trajectory.

**Table 3:** Multivariable logistic regression models of associations of sociodemographic and clinical characteristics with of fatigue (multinomial) and dyspnoea (binary) trajectory membership

	Fatigue			Dyspnoea
	Improving trajectory	Low-severe trajectory	High-severe trajectory	Severe trajectory
	RRR [95%CI]			OR [95%CI]
Age in years	0.99 [0.97; 1.02]	0.97 [0.95; 1.00]	0.98 [0.95; 1.01]	1.03 [1.00; 1.05]*
Sex (ref. = male)	1.78 [0.95; 3.34]	1.78 [0.91; 3.51]	2.45 [1.19; 5.05]*	1.41 [0.87; 2.29]
BMI (ref. = normal weight)				
Overweight	2.14 [1.12; 4.06]*	13.68 [0.81; 3.46]	0.94 [0.41; 2.15]	0.97 [0.50; 1.93]
Obese	4.29 [1.69; 10.85]**	4.76 [1.79; 12.66]**	6.59 [2.39; 18.14]***	3.04 [1.55; 6.18]**
Education (ref. = low/medium)	0.57 [0.32; 1.01]	0.38 [0.20; 0.73]**	0.52 [0.25; 1.07]	0.44 [0.23; 0.79]**
Comorbidity (ref. = none) ^a^	1.15 [0.63; 2.11]	3.38 [1.61; 7.10]**	3.68 [1.55; 8.76]**	
Respiratory condition (ref. = none)				6.07 [3.73; 10.02]***
At least one other comorbidity (ref. = none)				1.64 [0.94; 2.94]

*Notes*. ‘No symptom’ trajectory classes of fatigue and dyspnoea were set as reference outcome categories. ^a^ For dyspnoea, respiratory condition was assessed separately from other comorbidities. * *p* < 0.05; ** *p* < 0.01; *** *p* < 0.001. BMI = body mass index; CI = confidence intervals; OR = odds ratio; ref. = reference category; RRR = relative risk ratio

### Dyspnoea trajectories

The majority of participants (75%, *n* = 376) never or only once reported severe dyspnoea (‘no dyspnoea’ trajectory), while 25% (*n* = 123) experienced dyspnoea more often and were labelled as ‘severe’. One participant never filled out the mMRC and was left out of the analyses.

Compared to the ‘no dyspnoea’ trajectory, older age, being obese, having a low or medium education, and having a pre-existing respiratory condition were associated with being in the ‘severe’ trajectory.

### Overlap between trajectories

In total 51 combinations of trajectories (with between 1 (0.1%) to 57 (11.4%) included participants) were identified. Table 4 presents the 16 most common combinations of trajectories – defined as having at least 10 members – accounting for 72.8% of the sample.

**Table 4:** Most common combinations of identified trajectories ranked by frequency

	Trajectories				*n*	%
	Physical HRQoL	Mental HRQoL	Fatigue	Dyspnoea		
1	Improving	Stable good	Improving	No dyspnoea	57	11.4
2	Stable good	Stable good	No fatigue	No dyspnoea	44	8.8
3	Stable low	Low	High-severe	Severe dyspnoea	34	6.8
4	Stable low	Stable good	Improving	No dyspnoea	32	6.4
5	Improving	Improving	Improving	No dyspnoea	29	5.8
6	Improving	Stable good	No fatigue	No dyspnoea	26	5.2
7	Stable low	Low	Low-severe	No dyspnoea	22	4.4
8	Improving	Middle declining	Improving	No dyspnoea	20	4.0
9	Improving	Low	Low-severe	No dyspnoea	15	3.0
10	Stable good	Stable good	Improving	No dyspnoea	15	3.0
11	Improving	Low	Improving	No dyspnoea	13	2.6
12	Stable low	Low	Low-severe	Severe dyspnoea	13	2.6
13	Improving	Middle declining	Low-severe	No dyspnoea	12	2.4
14	Stable low	Middle declining	Low-severe	Severe dyspnoea	12	2.4
15	Stable low	Middle declining	High-severe	Severe dyspnoea	10	2.0
16	Stable low	Stable good	Low-severe	Severe dyspnoea	10	2.0

*Notes*. Percentages based on total sample (*N* = 500). HRQoL = health-related quality of life

The majority of these combinations includes participants that are still recovering (e.g. combinations 1 and 5) or still overall unrecovered (combinations 3 and 7), and only 8.8% seem to be fully recovered in terms of HRQoL and fatigue and dyspnoea symptoms (combination 2). Accordingly, many combinations show similar tendencies in both HRQoL domains. However, combinations such as 4, 8, and 9 show that physical and mental HRQoL can develop differently over time: low physical HRQoL does not necessarily mean low mental HRQoL for all participants.

Generally, worse HRQoL trajectories are consistent with severe symptom trajectories (e.g. combinations 3, 14 and 15) and vice versa (e.g. combinations 1, 2, and 5). The association of symptoms can however be different for each domain, as shown with combinations 9 (severe fatigue and worse mental HRQoL) and 16 (severe fatigue and dyspnoea, but good mental HRQoL).

## Discussion

Multiple trajectories of HRQoL and COVID-19-related symptoms were identified over the course of one year follow-up in patients that had been hospitalised with COVID-19. For physical HRQoL, three mostly stable trajectories were found, of which the vast majority of participants had a score consistently below the population norm. For mental HRQoL, four trajectories were identified, of which two classes (‘low’ and ‘middle declining’; in total 43%) had below-average scores. Associated factors were inconsistent between trajectories of physical and mental HRQoL. This suggests that researchers and healthcare professionals should not assume that there are fixed risk factors for poor long-term health outcomes, but that trajectories after COVID-19 can strongly differ per patient and HRQoL domain. Moreover, while physical and mental HRQoL domains are consistently either mostly good or worse, trajectories can differ for patients. Finally, severity of fatigue and dyspnoea symptoms seem to play a role in HRQoL after COVID-19.

The findings suggest that the development of physical HRQoL in COVID-19 survivors is at least partly associated with their health status before hospitalisation. That is, older age, overweight and obesity, and comorbidities were associated with worse HRQoL and vice versa for good HRQoL. Moreover, presence of severe fatigue and dyspnoea – be it from comorbidity or post-COVID-19 syndrome – is associated with lower physical HRQoL. A recent study similarly observed three distinct HRQoL trajectories in hospitalised Canadian COVID-19 survivors using the EQ-5D-5 L index [29]. Comparing the 3-to-12-month outcomes in both studies, similar longitudinal patterns can be observed, although the class sizes differed substantially. For example, Tanguay and colleagues found 28% classified as the lowest trajectory versus 44% in the present study [29]. This may partly be explained by differences in the EQ-5D-5 L index used by them (which also includes a mental HRQoL component) and the SF-36 PCS. Their findings suggested a continuous, strong decline after 12 months. Additionally, persistent reduced HRQoL and severe (post-COVID-19-related) symptoms were observed after 12 months [9]. Thus, further investigation and continued follow-up by clinicians after one year after hospital discharge are warranted in COVID-19 survivors with an already poor health status.

A worrying 26% of COVID-19 survivors consistently reported very poor mental HRQoL over the course of a year, with scores indicative of severe psychological symptoms [19]. This trajectory could not be explained by self-reported pre-existing comorbidity, including pre-existing psychiatric disorders. Psychological symptoms, especially depression, anxiety and post-traumatic stress disorder are commonly reported in the first three months after COVID-19-related hospital discharge and may persist for years [12]. A recent meta-analysis found that one in five COVID-19 survivors (22% pooled prevalence) continue to experience serious mental health problems for at least eight months [30]. As these problems were already identifiable after 12 weeks, early detection of and intervention in low mental HRQoL is strongly recommended, especially when patients also present with other post-COVID-19-related symptoms. An additional 17% declined after three months, warranting continuous monitoring. Somatic symptoms such as moderate or severe fatigue or dyspnoea may be associated with this decline, although evidence for that was uncertain in this study. Furthermore, patients may experience increasing difficulty with coping with their complaints for a prolonged time, uncertainty about (full) recovery, and increased pressure to return to work and other social roles [31–34].

Both severe fatigue (45% of participants) and severe dyspnoea (25%) were reported consistently over the course of a year and overlapped with low(er) HRQoL trajectories. These symptoms may (partly) stem from various (pre-existing) comorbidities, but seem also to be indicative of post-COVID-19 syndrome. For example, despite it being associated with higher odds for falling in the ‘severe’ dyspnoea trajectory, 44% of those 123 participants had no pre-existing respiratory condition (supplementary Table S8). Respiratory complications, such as pulmonary fibrosis, are common in COVID-19 survivors and are associated with post-COVID-19 syndrome [35]. The long-lasting detrimental effects on both physical and mental HRQoL, often paired with persistent fatigue and dyspnoea, suggest a potential disruption in the body’s normal recovery process after the initial acute infection. This may be indicative of lasting damage caused by ongoing immune activation and chronic inflammation, as postulated for post-COVID-19 syndrome [36].

One surprising finding was that the trajectories showed relatively little variation over time: no substantial (drastic) improvements or declines in HRQoL or symptom severity at specific times were observed. This finding seems to imply that interventions for approving HRQoL may be initiated as soon as 12 weeks after the initial SARS-CoV-2 infection if patients indicate below-average or low well-being and functioning. However, further research, for example using intensive longitudinal research designs such as experience sampling methodology, is required to ascertain this. Such designs include continuous or repeated measurements over a short time, such as 10 times a day for 14 consecutive days, providing rich insights into the daily life experiences of patients [37].

The over 16 identified combinations of HRQoL and symptom trajectories showcase the diversity of needs patients may have after COVID-19. Some patients may benefit from psychological interventions, such as using Cognitive Behavioural Therapy or Acceptance-Commitment Therapy [38, 39], while others may be better helped with (multidisciplinary) interventions targeting somatic aspects, such as pharmacotherapy, physiotherapy, and occupational therapy [39, 40], and may additionally benefit from self-management interventions (e.g. self-monitoring and energy management support or emotion regulation skills) [41]. Clinicians could use HRQoL measurements to assess these needs, along with patient wishes, to identify different care options suitable to the patient.

Regular measurements of both physical and mental HRQoL are recommended to identify those who will benefit from interventions at the right time. Further identifying patterns in symptom persistence (such as symptoms clusters [4, 5]), patient demographics, and biochemical profiles is crucial to understand the different HRQoL trajectories. For example, clinical characteristics such as hospital-received drug treatment during and after hospitalisation, disease severity and intensive care admission may play a role in HRQoL trajectories, as may sociodemographic and sociopsychological factors such as income, ethnicity, occupational situation, access to care, and social support. For biochemical profiles, measuring C-reactive protein provides an indication of inflammation and has been used to evaluate disease severity and mortality in COVID-19 patients [42, 43]. Alternatively, measuring pro- and anti-inflammatory cytokines, such as interleukin-6 and − 10, tumour necrosis factor-alpha, and interferon-gamma identify patients with an immune dysregulation which may manifest more prominently as the disease progresses [44, 45]. This may allow for more timely initiation of medical interventions limiting bodily damage and improving HRQoL development. Additionally, assessing these factors helps narrow down potential risk factors and pathophysiological mechanisms, and is essential in developing personalised interventions. Similarly, intensive longitudinal studies can explore relationships between persisting symptoms, well-being and daily life functioning [46, 47]. Meanwhile, telemonitoring can help with continuous screening and earlier detection of complications while relieving clinicians [48, 49].

Caution should be taken in generalising the study findings to patients that were not hospitalised. As Tanguay and colleagues [29] have shown, HRQoL trajectories differ for ex-hospitalised and non-hospitalised COVID-19 survivors. It is unclear to what extent the results can be generalised to subgroups that experienced difficulties gaining access to (hospital) healthcare [50–52]. The inclusion criterion of Dutch proficiency may have resulted in a selection bias. Previously hospitalised patients with a migrant background have a higher risk of developing post-COVID-19 syndrome compared to Dutch origin patients [53], suggesting a likelihood of worse HRQoL in migrant subpopulations. These subgroups may not have been captured in the current study. Future studies are recommended to assess, and – if necessary – account for these sociodemographic differences.

### Strengths and limitations

The current study was – to our knowledge – the first study to assess heterogeneity of multiple HRQoL and symptom severity trajectories in COVID-19 survivors. It was executed in COVID-19 patients of two hospitals covering a large region in the Netherlands, using well-validated, standardised questionnaires to measure HRQoL, fatigue and dyspnoea. The findings are comparable to those of cross-sectional or longitudinal studies in similar populations, which suggest that the established trajectories can be helpful for considering factors in heterogeneity of HRQoL development in COVID-19 survivors, as well in considering targets and timing of related interventions. However, GMM is a data-driven approach, and prediction of classification was not the main objective of this study, so generalisations should be made with care. Nevertheless, studies in other countries found comparable results [8, 29].

Some limitations should be addressed. The entropy indices of the included models were lower than preferable (> 0.8), suggesting reduced accuracy. However, the posterior probability of classifications showed that on average, trajectories were at least adequate (> 0.8), indicating that while there is some level of uncertainty about the classification of some individuals, there should be few class membership errors. Nevertheless, because of the lower entropy values and the utilised standard three-step method, associations between class membership and covariates may be underestimated [54]. At the time of analysis, *lcmm* provided no means to adjust for classification uncertainty.

Another limitation was that clinical data were collected using self-reported items in broad categories and open for the interpretation, recollection, and social biases of participants, possibly leading to both over- and underreporting of comorbidities (e.g. psychiatric disorders). Also, the nature, severity, and timeline of comorbidities could not be accounted for in the analyses. Consequently, it might be the case that two minor temporary conditions (e.g. urinary tract infection and a mild sport injury) are weighted stronger than one major comorbidity (e.g. lung cancer) if only frequency of presence is investigated. To counteract this risk, comorbidity was dichotomised as present or absent in the regression analyses.

Additionally, some potentially beneficial information in retrospective was not anticipated in the study design in April 2020, or could not (accurately) be retrieved (e.g. the type of SARS-CoV-2 and number of vaccinations) from the health records. Assessing these factors and other clinical, sociodemographic and sociopsychological factors, as well as continuous observations after 12 months, is recommended for future studies and could result in improved risk profiles.

Furthermore, while it is common to assume that data are missing at random, it might be the case that participants dropped out due to feeling too unwell to participate (i.e. low HRQoL) or due to fully recovering from COVID-19 (i.e. high HRQoL) and not feeling incentivised to participate. However, drop-out analysis showed no evidence for this. Still, reasons for non-participation are unknown and certain (self-)selection biases, including proficiency in Dutch, may limit generalisability. Similarly, excluding patients with less than 3 out of 4 measurements may have affected the generalisability of the findings.

Finally, as there are no recent representative Dutch norms available for the SF-36, most Dutch studies use the US-based general population norms to compute the summary scores [55]. It is currently unclear to what extent these norms are exactly applicable to the Dutch population, which should be kept in mind when interpreting absolute values of these scores. Moreover, we have assumed that the measurement structure of the outcomes are invariant over time. Previous studies have found the SF-36 to be longitudinally invariant in various populations [e.g. 56, 57]. Nevertheless, this should be evaluated in hospitalised COVID-19 survivors.

## Conclusion

The current study found that the longitudinal courses of health-related quality of life vary between hospitalised COVID-19 survivors; should be distinguished between physical and mental domains; and seem to be associated with the severity of post-COVID-19-related symptoms, specifically with the courses of fatigue and dyspnoea. Regular measurement of HRQoL and post-COVID-19 symptoms may help identify those patients that may benefit from timely interventions. Interventions may be initiated as early as 12 weeks after infection, especially in patients with risk factors. Future studies should validate these findings and assess how these courses further develop after one year.

## Electronic supplementary material

Below is the link to the electronic supplementary material.

Supplementary Material 1

## Data Availability

The data are not publicly available. Enquiries for data access upon reasonable request should be directed to h.vonkeman@mst.nl.
