# Supplementary material for: One-year trajectories of physical and mental health-related quality of life, fatigue and dyspnoea in COVID-19 survivors
Source: Qual Life Res. 2024 Oct 19;34(2):341–51. doi: 10.1007/s11136-024-03812-y (PMC11865161; doi:10.1007/s11136-024-03812-y)
Supplement: Supplementary file 1 — Supplementary Material 1 [file 11136_2024_3812_MOESM1_ESM.pdf]

One-year trajectories of physical and mental health-related quality of life, fatigue and dyspnoea in COVID-19 survivors

Supplementary Material

Contents

Model selection procedure and summary ... **2**

Model selection physical HRQoL trajectories ... **3**

Supplementary Table S1 Fit indices for investigated models of physical HRQoL trajectories ... 3

Fig. S1 Linear LCGA class trajectories of latent classes in physical HRQoL (PCS) ... 4

Fig. S2 Quadratic LCGA class trajectories of latent classes in physical HRQoL (PCS) ... 4

Fig. S3 Linear GMM class trajectories of latent classes in physical HRQoL (PCS) ... 5

Fig. S4 Quadratic GMM class trajectories of latent classes in physical HRQoL (PCS) ... 5

Model selection mental HRQoL trajectories ... **6**

Supplementary Table S2 Fit indices for investigated models of mental HRQoL trajectories ... 6

Fig. S5 Linear LCGA class trajectories of latent classes in mental HRQoL (MCS) ... 7

Fig. S6 Quadratic LCGA class trajectories of latent classes in mental HRQoL (MCS) ... 7

Fig. S7 Linear GMM class trajectories of latent classes in mental HRQoL (MCS) ... 8

Fig. S8 Quadratic GMM class trajectories of latent classes in mental HRQoL (MCS) ... 8

Model selection fatigue trajectories ... **9**

Supplementary Table S3 Fit indices for investigated models of fatigue trajectories ... 9

Fig. S9 Linear LCGA class trajectories of latent classes in fatigue (SFQ) ... 10

Fig. S10 Quadratic LCGA class trajectories of latent classes in fatigue (SFQ) ... 10

Fig. S11 Linear GMM class trajectories of latent classes in fatigue (SFQ) ... 11

Fig. S12 Quadratic GMM class trajectories of latent classes in fatigue (SFQ) ... 11

Dyspnoea trajectories ... **12**

Supplementary Table S4 Frequencies of dyspnoea severity ... 12

Fig. S13 Alluvial plot showing the flow of dichotomised dyspnoea scores (severe/not severe) ... 12

Drop-out analysis ... **13**

Supplementary Table S5 Characteristics of included and dropped-out participants ... 13

Distributions of outcome measures ... **14**

Supplementary Table S6 Distribution of observed outcomes by measurement points and trajectory ... 14

Trajectories of individuals per outcome and class ... **15**

Fig. S14 Individual trajectories of physical HRQoL (PCS) ... 15

Fig. S15 Individual trajectories of mental HRQoL (MCS) ... 16

Fig. S16 Individual trajectories of fatigue (SFQ) ... 17

Characteristics per outcome trajectories ... **18**

Supplementary Table S7 Characteristics of health-related quality of life trajectories ... 18

Supplementary Table S8 Characteristics of fatigue and dyspnoea trajectories ... 19

R syntax ... **20**

GRoLTS Checklist ... **34**

STROBE Checklist ... **35**

Model selection procedure and summary

Growth Mixture Modelling (GMM) was applied to investigate unobserved heterogeneity in development trajectories of physical and mental health-related quality of life (HRQoL), and fatigue severity, while modelling dyspnoea severity was found to not be feasible (see p. 12 of this document). To determine the most suitable model structures, multiple aspects were considered: 1) the number of latent classes; 2) the shape and functional form of the trajectories: linear or quadratic; 3) specification of within-class heterogeneity (i.e. LCGA (Latent Class Growth Analysis) or GMM); 4) the structure of the variance-covariances matrices (i.e. across-class heterogeneity); and 5) random intercept-only or random intercept and slopes models for GMM.

Overall, LCGA (restricted within-class variance, i.e. assumed within-class homogeneity) performed worse compared to GMM (within-class heterogeneity[1][1]). Preliminary investigations showed that for both linear and quadratic functions, the unstructured (i.e. freely estimated) variance-covariance structure models performed better in terms of model indices and showed no issues with convergence. Likewise, the GMM that included both random intercepts and slopes were found to be most informative. The *R* syntax is presented at pp. 20 – 33 of this document.

The optimal number of trajectory classes was determined via statistical criteria and clinical interpretability. The lowest Bayesian Information Criterion (BIC) value was primarily used to identify the best fit, but sample size-adjusted BIC (saBIC) and Akaike Information Criterion (AIC) indices were also considered. The accuracy of the classification was evaluated using the entropy index, with values closer to 1 representing a higher adequacy. Solutions were subsequently plotted and analysed visually for clinical meaningfulness. Finally, the average, lowest and highest values of the posterior probability (i.e. likelihood of an individual to be assigned to a trajectory) of the included classes were reported. The relevant statistical criteria are reported in Table S1 for physical HRQoL, Table S2 for mental HRQoL, and Table S3 for fatigue. The related class trajectories are reported in Fig. S1 – S4 for physical HRQoL, Fig. S5 – S8 for mental HRQoL, and Fig. S9 – S12 for fatigue.

Quadratic GMM were favoured for all outcomes over LCGA and linear GMM structures (Tables S1 – S3). A 3-classes solution was deemed favourable for physical HRQoL based on BIC and clinical relevance. For mental HRQoL, statistical arguments for both three classes (based on BIC) and four classes (saBIC and AIC) could be made. Based on visual assessment (Fig. S8), the extra class was considered to lead to improved clinical insights; hence, the 4-classes solution was preferred. Similarly, both four classes and five classes were probable for fatigue, but the additional class was not considered as clinically relevant, resulting in a favourable 4-classes solution. Individual trajectories within each class are presented in Fig. S14 – S16.

[1] [1] van de Schoot, R., Sijbrandij, M., Winter, S. D., Depaoli, S., & Vermunt, J. K. (2017). The GRoLTS-Checklist: Guidelines for Reporting on Latent Trajectory Studies. *Structural Equation Modeling: A Multidisciplinary Journal*, *24*(3), 451-467. https://doi.org/10.1080/10705511.2016.1247646

Model selection physical HRQoL trajectories

Supplementary Table S1 Fit indices for investigated models of physical HRQoL trajectories

	BIC	saBIC	AIC	Entropy	Class sizes (%)	PP [a]
**LCGA Linear**						
1 class	13336.08	13323.38	13319.22	1.00	100	-
2 classes	12429.18	12403.79	12395.47	0.88	52.4; 47.6	0.97 (0.96 – 0.97)
3 classes	12210.53	12172.44	12159.95	0.84	35.2; 34.0; 30.8	0.93 (0.89 – 0.95)
4 classes	12143.91	12093.12	12076.47	0.80	29.0; 27.6; 28.6; 14.8	0.89 (0.87 – 0.93)
5 classes	12138.28	12074.80	12053.99	0.78	29.0; 28.0; 19.8; 14.6; 8.6	0.84 (0.75 – 0.92)
**LCGA Quadratic**						
1 class	13336.08	13323.38	13319.22	1.00	100	-
2 classes	12429.18	12403.79	12395.47	0.88	52.4; 47.6	0.97 (0.96 – 0.97)
3 classes	12210.53	12172.44	12159.95	0.84	35.2; 34.0; 30.8	0.93 (0.89 – 0.95)
4 classes	12143.91	12093.12	12076.47	0.80	29.0; 27.6; 28.6; 14.8	0.89 (0.87 – 0.93)
5 classes	12138.28	12074.80	12053.99	0.74	29.0; 28.0; 19.8; 14.6; 8.6	0.84 (0.75 – 0.92)
**GMM Linear**						
1 class	12126.19	12107.14	12100.90	1.00	100	-
2 classes	12083.00	12051.26	12040.86	0.76	53.4; 46.6	0.93 (0.93 - 0.93)
3 classes	12071.55	12027.11	12012.54	0.70	50.0; 29.6; 20.4	0.84 (0.78 - 0.92)
4 classes	12076.94	12019.81	12001.08	0.77	53.4; 23.8; 21.8; 1.0	0.83 (0.78 - 0.92)
5 classes	12089.95	12020.15	12089.95	0.76	44.8; 23.8; 20.6; 9.6; 1.2	0.81 (0.65 - 0.92)
**GMM Quadratic**						
1 class	12118.33	12086.59	12076.19	1.00	100	-
2 classes	12059.75	12012.14	11996.53	0.79	54.4; 45.6	0.94 (0.93 - 0.95)
3 classes	**12040.83**	**11977.35**	11956.54	0.74	44.0; 40.2; 15.8	0.86 (0.79 - 0.92)
4 classes	12056.86	11977.51	11951.49	0.70	41.6; 25.6; 17.0; 15.8	0.76 (0.64 - 0.87)
5 classes	12068.58	11973.36	**11942.14**	0.69	40.4; 24.0; 16.2; 10.2; 9.2	0.78 (0.71 - 0.87)

Notes. AIC = Akaike Information Criterion, BIC = Bayesian Information Criterion, GMM = growth mixture modelling, LCGA = latent class growth analysis, saBIC = Sample-size Adjusted Bayesian Information Criterion. [a] PP = Average of posterior probability of the model with min and max values in parentheses. Models fitted with 100 random starts for all LCGA and the GMM 1 and 2 classes, and were increased to 500 random starts for the GMM 3 – 5 classes models. Values in **bold** indicate statistically best fit.

Fig. S1 Linear LCGA class trajectories of latent classes in physical HRQoL (PCS)

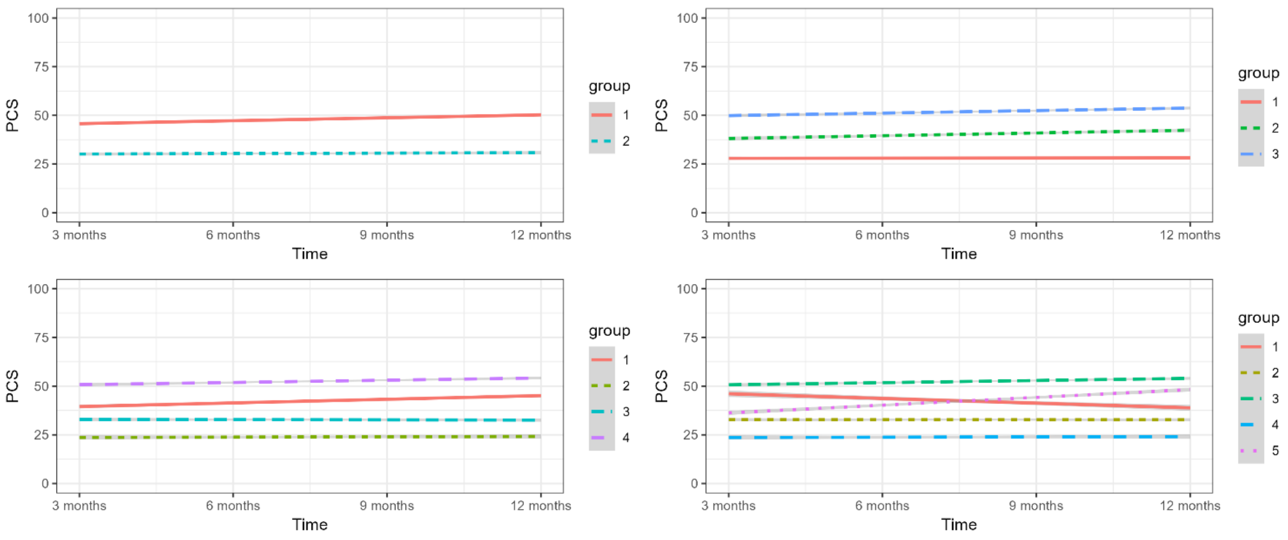

Fig. S2 Quadratic LCGA class trajectories of latent classes in physical HRQoL (PCS)

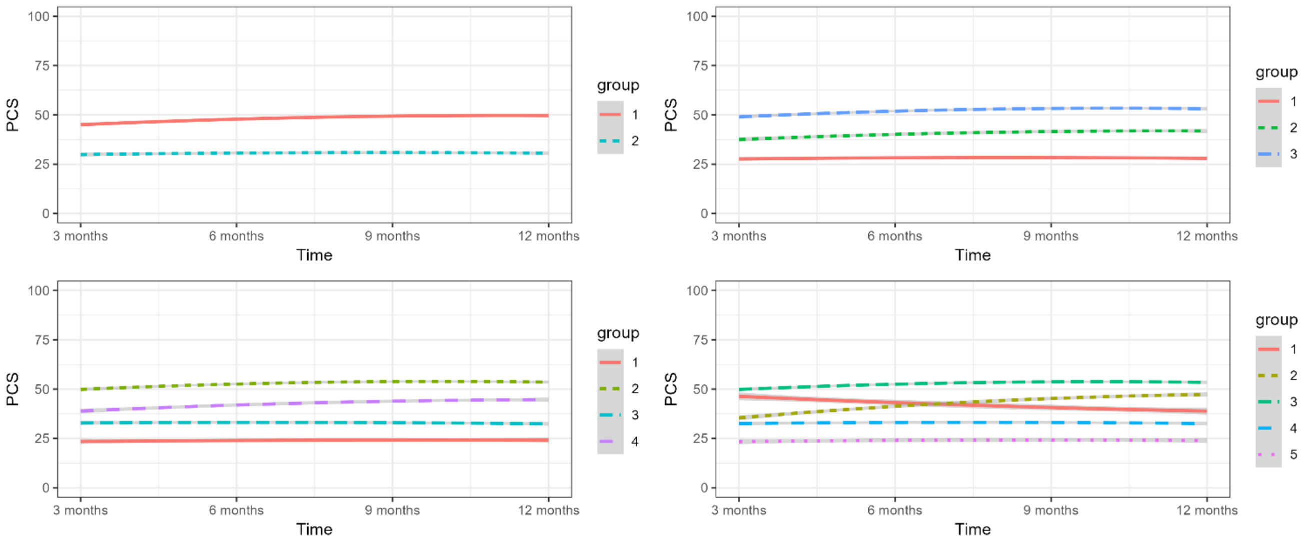

Fig. S3 Linear GMM class trajectories of latent classes in physical HRQoL (PCS)

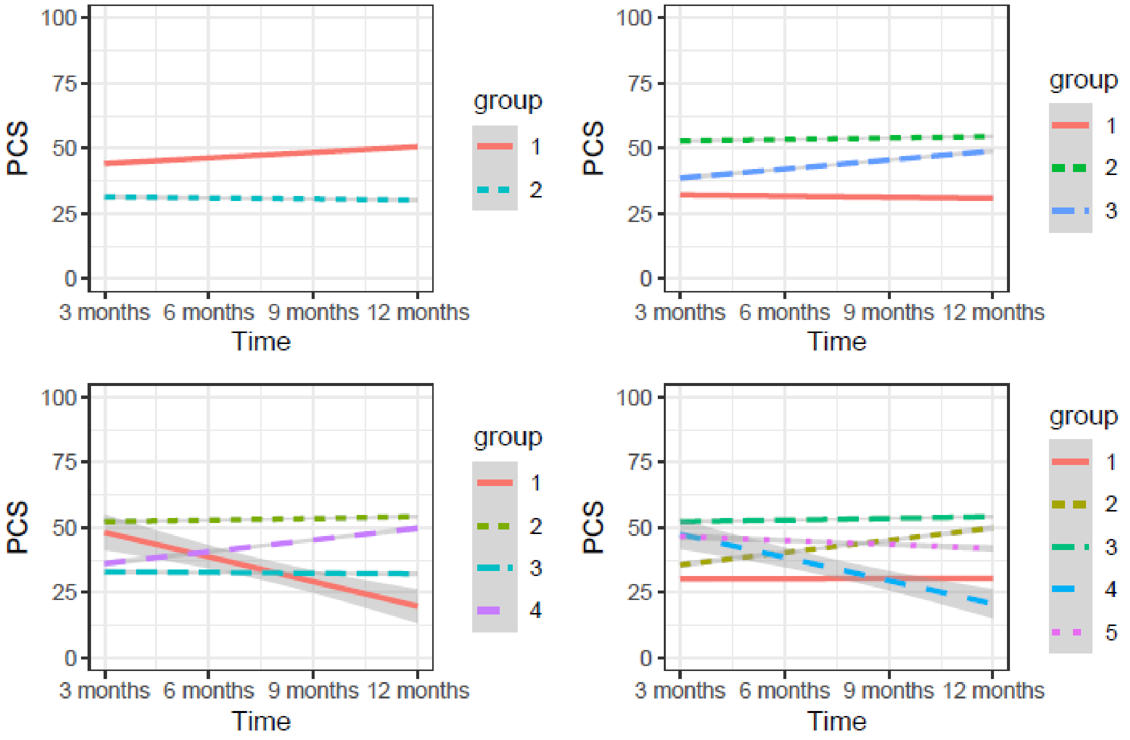

Fig. S4 Quadratic GMM class trajectories of latent classes in physical HRQoL (PCS)

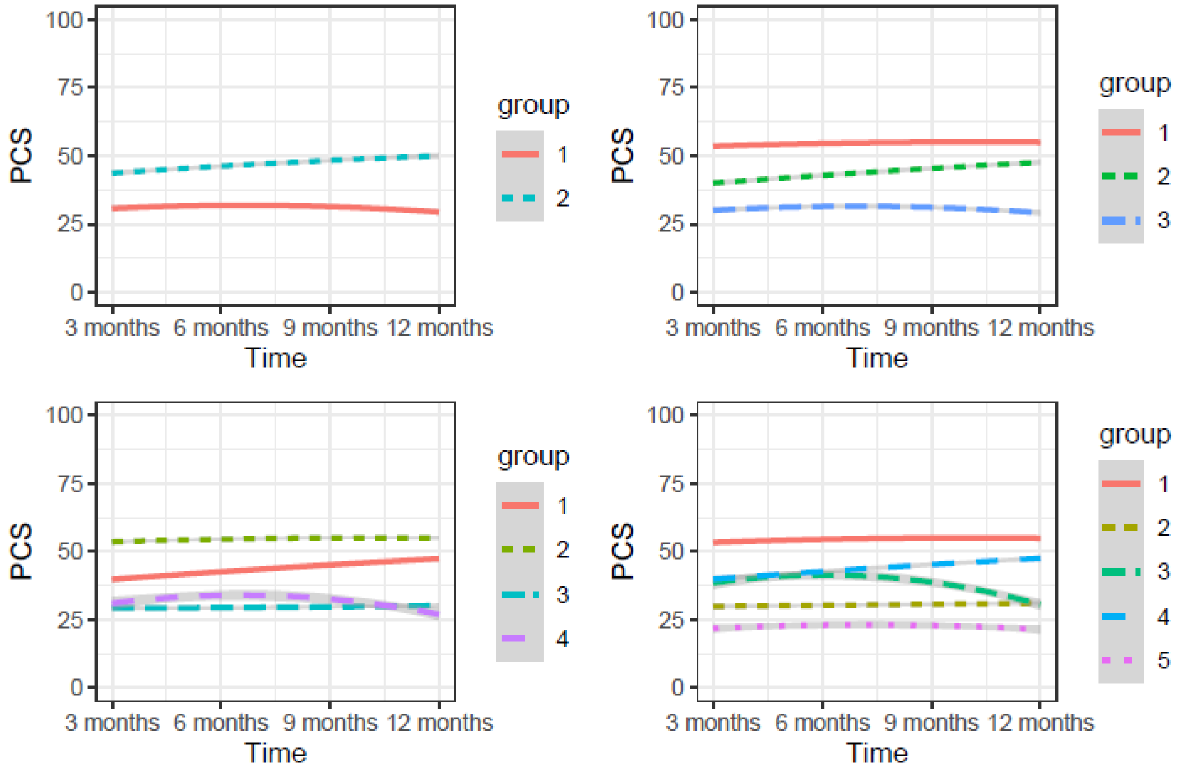

Model selection mental HRQoL trajectories

Supplementary Table S2 Fit indices for investigated models of mental HRQoL trajectories

	BIC	saBIC	AIC	Entropy	Class sizes (%)	PP [a]
**LCGA Linear**						
1 class	12981.14	12971.62	12968.50	1.00	100	-
2 classes	12338.09	12319.05	12312.80	0.86	66.4; 31.6	0.96 (0.95 – 0.96)
3 classes	12201.22	12172.66	12163.29	0.82	51.4; 35.2; 10.4	0.91 (0.88 – 0.95)
4 classes	12174.18	12136.09	12123.60	0.76	45.6; 26.6; 21.0; 6.8	0.85 (0.77 - 0.91)
5 classes	12192.82	12145.21	12129.60	0.61	42.4; 29.8; 21.0; 6.8; 0	0.75 (0.56 – 0.85)
**LCGA Quadratic**						
1 class	12986.53	12973.83	12969.67	1.00	100	-
2 classes	12348.65	12323.26	12314.94	0.86	68.0; 32.0	0.96 (0.95 – 0.97)
3 classes	12217.71	12179.63	12167.14	0.83	54.4; 35.6; 10.0	0.91 (0.88 – 0.95)
4 classes	12184.31	12133.52	12116.87	0.77	47.6; 24.6; 19.8; 8.0	0.87 (0.76 – 0.93)
5 classes	12164.16	12100.68	12079.86	0.79	45.8; 22.8; 19.0; 6.6; 5.8	0.85 (0.78 – 0.92)
**GMM Linear**						
1 class	12243.09	12224.04	12217.80	1.00	100	-
2 classes	12141.24	12109.50	12099.10	0.63	55.2; 44.8	0.89 (0.83 - 0.94)
3 classes	12146.15	12101.72	12087.15	0.64	43.8; 38.6; 17.6	0.79 (0.60 - 0.90)
4 classes	12164.85	12107.71	12088.98	0.66	42.8; 29.8; 21.0; 6.4	0.78 (0.65 - 0.90)
5 classes [b]	12184.34	12114.51	12091.62	0.67	42.2; 23.8; 21.8; 6.4; 5.8	0.75 (0.67 - 0.91)
**GMM Quadratic**						
1 class	12263.18	12231.44	12221.04	1.00	100	-
2 classes	12104.26	12056.65	12041.04	0.71	56.8; 43.2	0.92 (0.87 - 0.96)
3 classes	**12095.41**	12031.93	12011.12	0.72	42.6; 42.6; 14.8	0.84 (0.70 - 0.93)
4 classes	12108.20	**12028.85**	**12002.83**	0.69	42.6; 26.0; 17.0; 14.4	0.80 (0.69 - 0.90)
5 classes	12129.21	12033.99	**12002.77**	0.74	37.6; 30.6; 15.0; 11.6; 5.2	0.78 (0.57 - 0.89)

Notes. AIC = Akaike Information Criterion, BIC = Bayesian Information Criterion, GMM = growth mixture modelling, LCGA = latent class growth analysis, saBIC = Sample-size Adjusted Bayesian Information Criterion. [a] PP = Average of posterior probability of the model with min and max values in parentheses. [b] Model did not converge. Models fitted with 100 random starts for all LCGA and the GMM 1 and 2 classes, and were increased to 500 random starts for the GMM 3 – 5 classes models. Values in **bold** indicate statistically best fit.

Fig. S5 Linear LCGA class trajectories of latent classes in mental HRQoL (MCS)

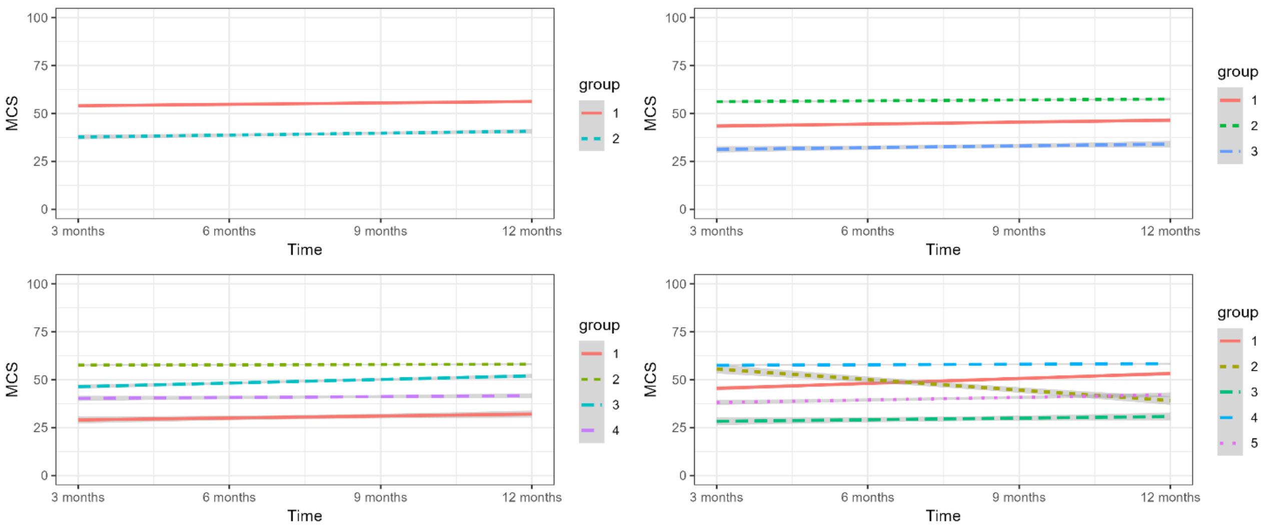

Fig. S6 Quadratic LCGA class trajectories of latent classes in mental HRQoL (MCS)

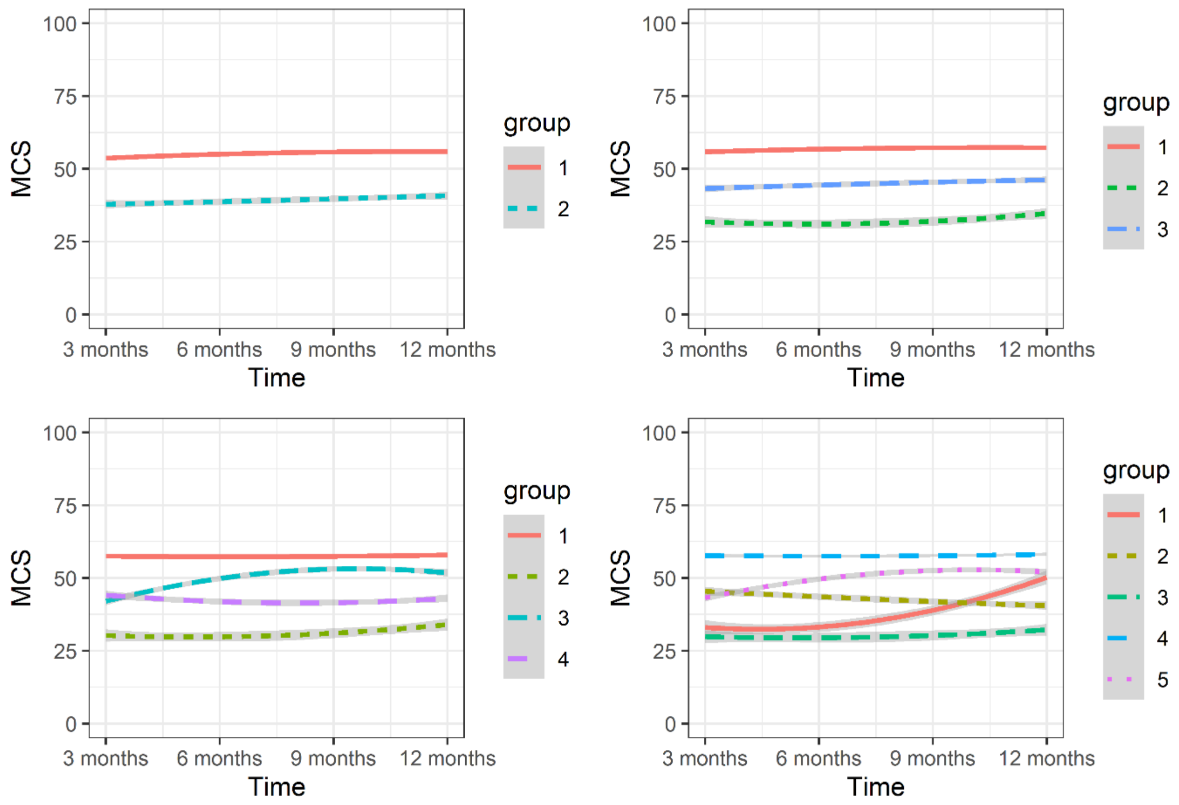

Fig. S7 Linear GMM class trajectories of latent classes in mental HRQoL (MCS)

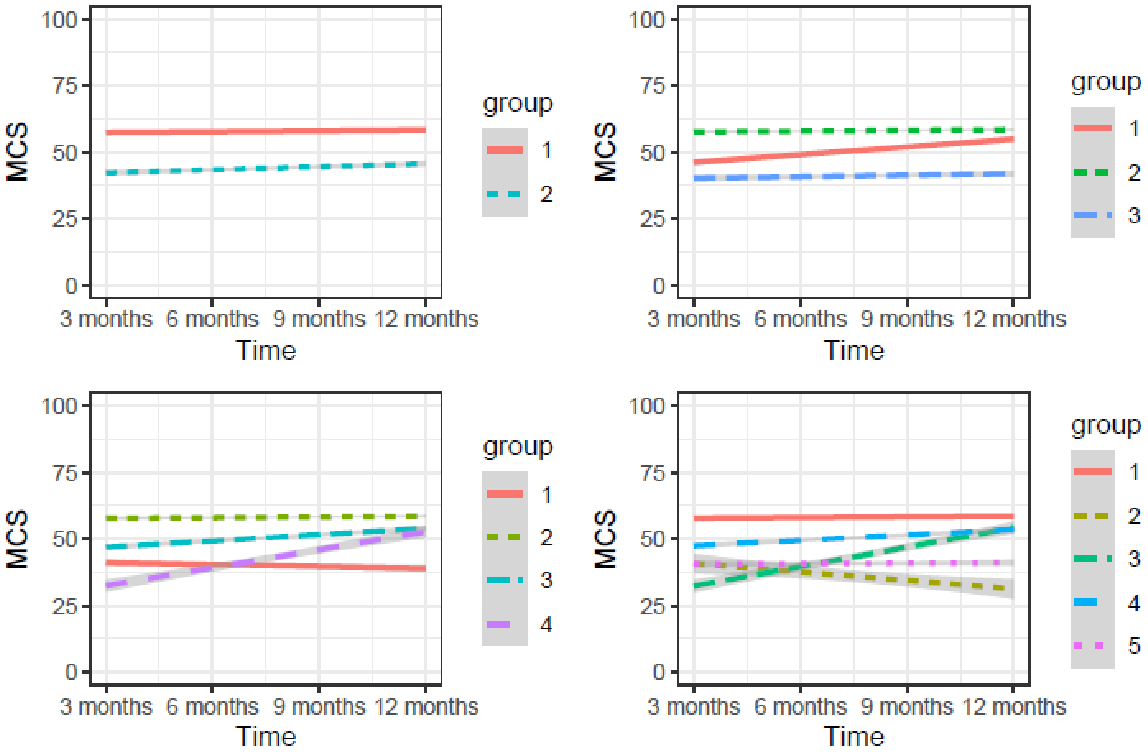

Fig. S8 Quadratic GMM class trajectories of latent classes in mental HRQoL (MCS)

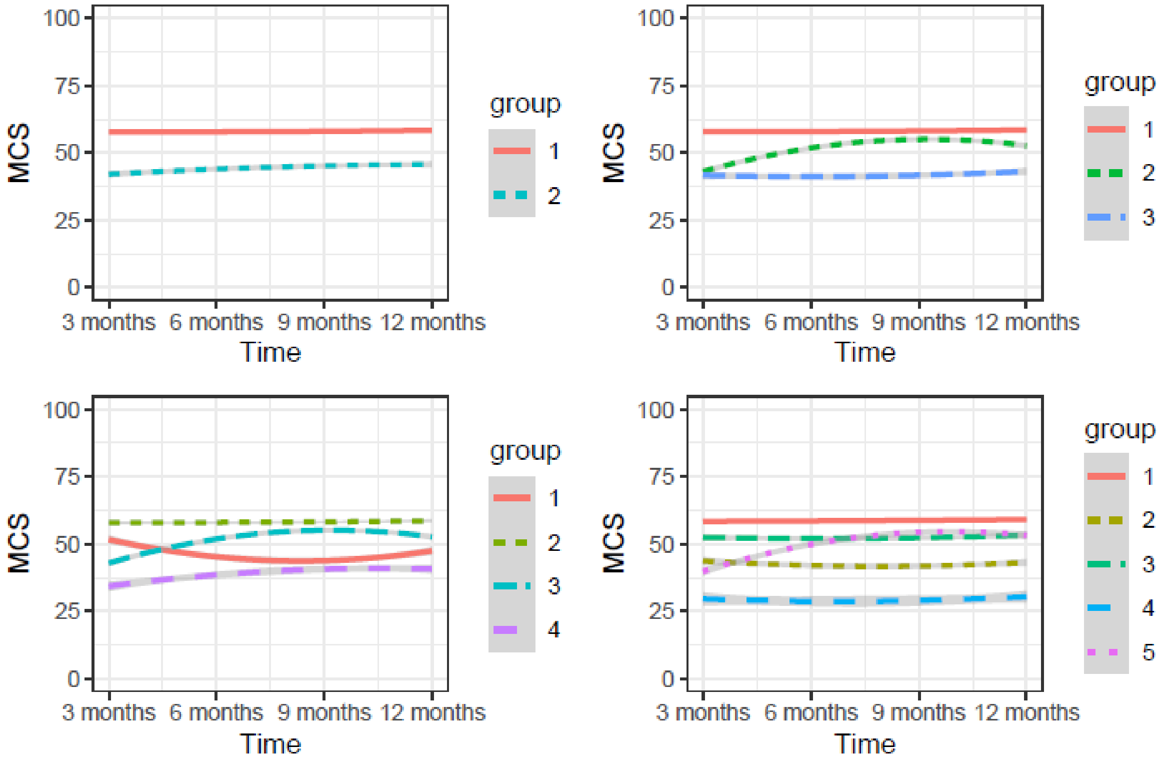

Model selection fatigue trajectories

Supplementary Table S3 Fit indices for investigated models of fatigue trajectories

	BIC	saBIC	AIC	Entropy	Class sizes (%)	PP [a]
**LCGA Linear**						
1 class	11494.11	11484.58	11481.46	1.00	100	-
2 classes	10500.28	10481.23	10474.99	0.90	60.4; 39.6	0.97 (0.97 – 0.97)
3 classes	10187.76	10159.19	10149.83	0.87	40.4; 37.2; 22.4	0.94 (0.89 – 0.96)
4 classes	10115.24	10077.15	10064.67	0.82	30.4; 29.8; 21.0; 18.8	0.90 (0.85 – 0.94)
5 classes	10073.89	10026.28	10010.67	0.82	31.6; 29.6; 18.4; 12.4; 8.0	0.87 (0.80 – 0.84)
6 classes	10051.77	9994.64	9975.91	0.79	23.4; 22.0; 18.2; 18.2; 9.8; 8.4	0.84 (0.79 – 0.93)
7 classes	10055.35	9988.70	9966.84	0.77	22.8; 18.2; 17.8; 17.6; 9.4; 8.8; 5.4	0.81 (0.70 – 0.94)
**LCGA Quadratic**						
1 class	11497.87	11485.17	11481.01	1.00	100	-
2 classes	10501.08	10475.69	10467.36	0.90	60.8; 39.2	0.97 (0.97 – 0.97)
3 classes	10195.35	10157.26	10144.77	0.86	40.4; 35.0; 24.6	0.94 (0.91 – 0.96)
4 classes	10119.78	10068.99	10052.35	0.82	30.8; 30.6; 20.4; 18.2	0.90 (0.85 – 0.95)
5 classes	10073.59	10010.11	9989.30	0.83	31.0; 29.2; 18.2; 11.8; 9.8	0.87 (0.80 – 0.94)
6 classes	10052.26	9976.09	9951.11	0.80	23.8; 21.6; 18.2; 17.6; 10.0; 8.8	0.84 (0.80 – 0.94)
7 classes [b]	10054.13	9965.29	9936.12	0.81	23.4; 22.4; 18.2; 17.6; 7.8; 7; 3.6	0.83 (0.73 – 0.93)
**GMM Linear**						
1 class	10129.93	10110.88	10104.64	1.00	100	-
2 classes	10068.82	10037.08	10026.67	0.64	57.8; 42.2	0.89 (0.84 - 0.94)
3 classes	10036.90	9992.46	9977.89	0.72	49.4; 35.0; 15.6	0.86 (0.82 - 0.92)
4 classes [c]	10042.67	9985.54	9966.81	0.73	42.8; 23.4; 18.2; 15.6	0.84 (0.73 - 0.91)
**GMM Quadratic**						
1 class	10132.01	10100.27	10089.86	1.00	100	-
2 classes	10061.35	10013.74	9998.13	0.66	60.0; 40.0	0.89 (0.84 - 0.94)
3 classes	10057.74	9994.25	9973.44	0.74	39.6; 30.2; 30.2	0.90 (0.85 - 0.97)
4 classes	**10012.35**	9933.00	9906.98	0.77	39.8; 26.6; 18.2; 15.4	0.87 (0.78 - 0.92)
5 classes	10018.04	**9922.82**	**9891.60**	0.81	23.2; 21.2; 22.0; 18.2; 15.4	0.86 (0.81 - 0.95)
6 classes [c]	10040.25	9929.16	9892.74	0.82	23.2; 22.2; 19.4; 18.2; 15.4; 1.6	0.85 (0.74 – 0.94)

Notes. AIC = Akaike Information Criterion, BIC = Bayesian Information Criterion, GMM = growth mixture modelling, LCGA = latent class growth analysis, saBIC = Sample-size Adjusted Bayesian Information Criterion. [a] PP = Average of posterior probability of the model with min and max values in parentheses. [b] 8-classes model was not fit as class sizes became too small for clinical relevance (<5%). [c] Model did not converge. Models fitted with 100 random starts for LCGA and GMM 1 and 2 classes, and were increased to 250 random starts for the quadratic LCGA and 1,000 random starts for the GMM 3 – 5 classes models. Values in **bold** indicate statistically best fit.

Fig. S9 Linear LCGA class trajectories of latent classes in fatigue (SFQ)

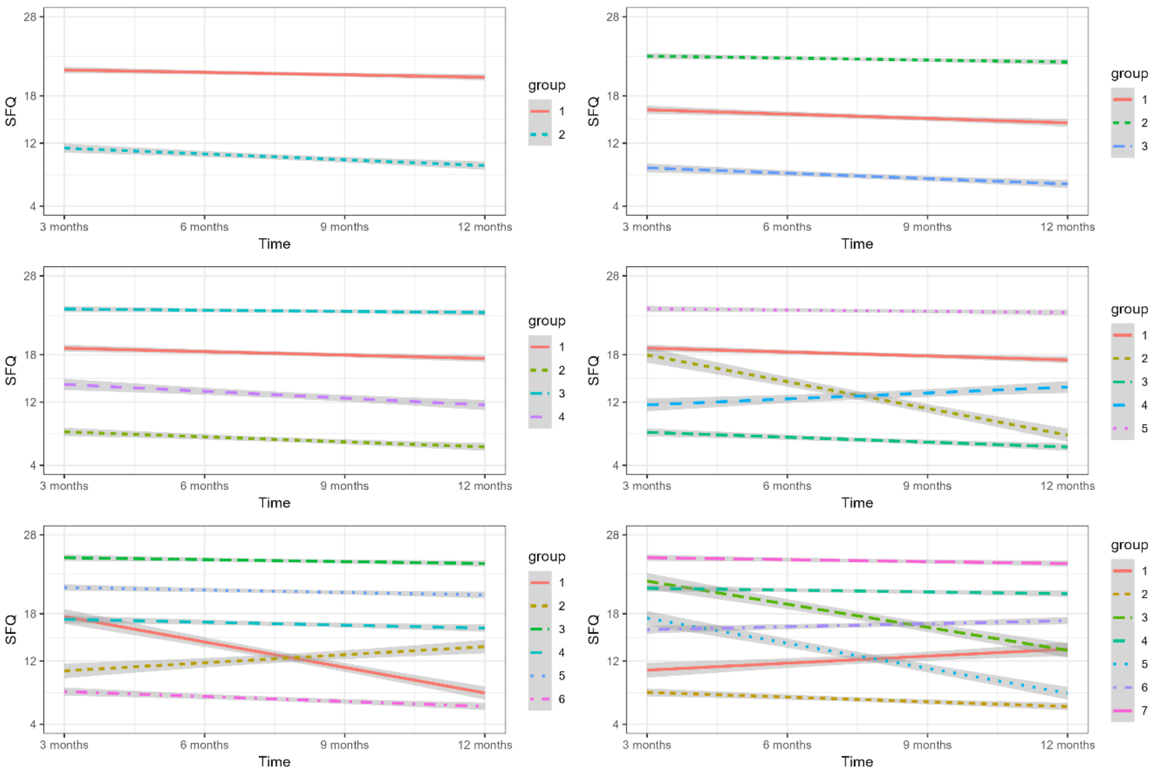

Fig. S10 Quadratic LCGA class trajectories of latent classes in fatigue (SFQ)

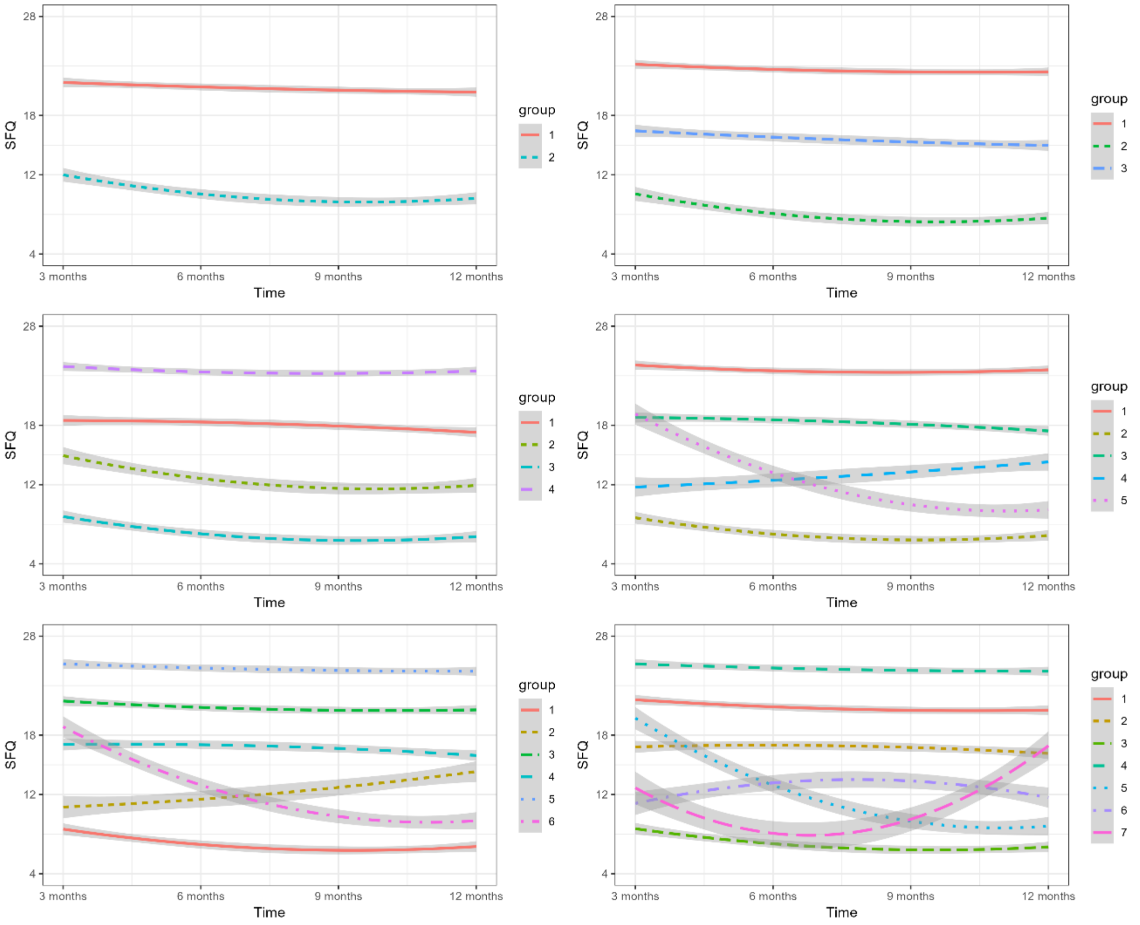

Fig. S11 Linear GMM class trajectories of latent classes in fatigue (SFQ)

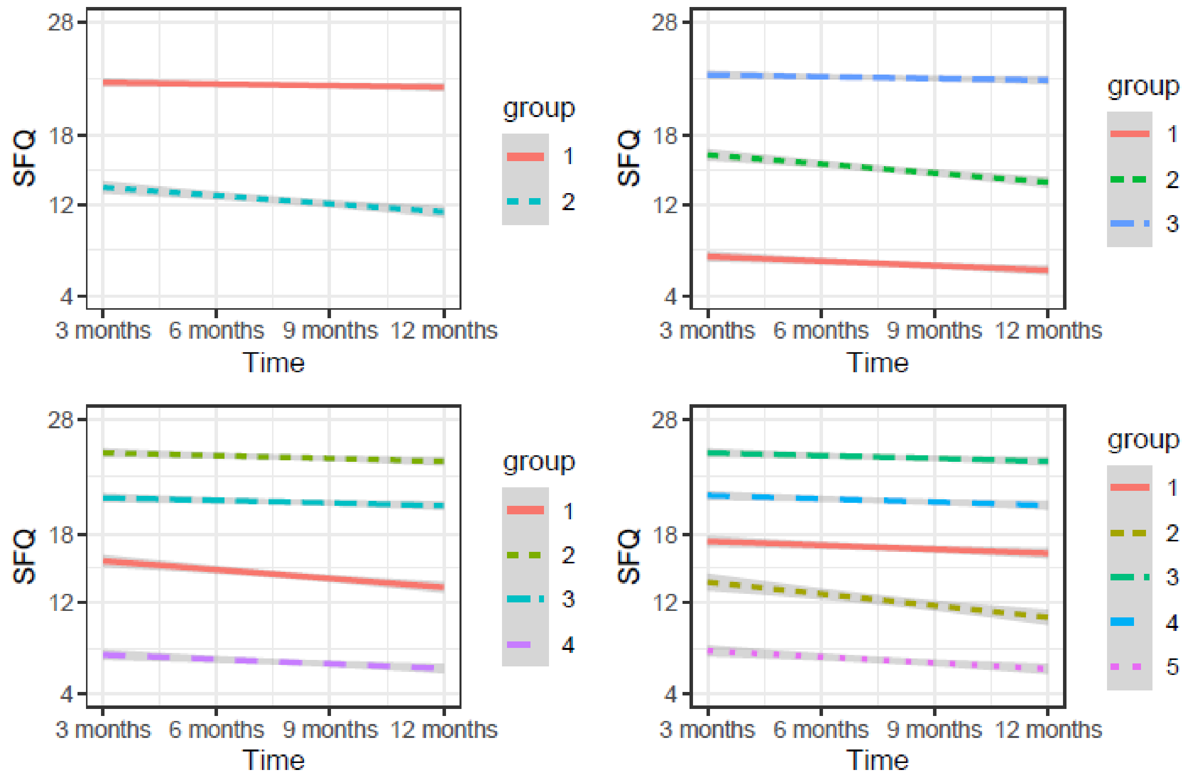

Fig. S12 Quadratic GMM class trajectories of latent classes in fatigue (SFQ)

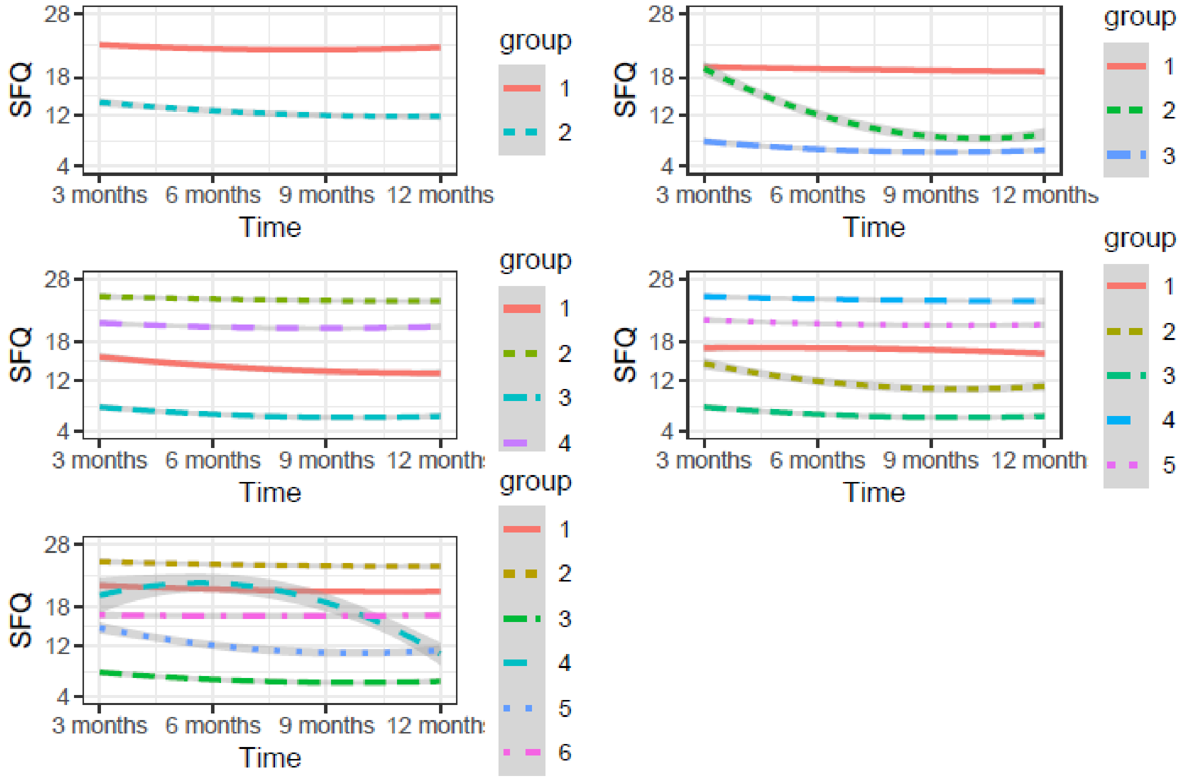

Dyspnoea trajectories

Exploratory analyses showed that dyspnoea severity, as measured by the mMRC, was mostly static in participants: over the course of the data collection period, they had either never or only at one measurement point a score above the cutoff for clinically severe dyspnoea (mMRC ≥2), or at two or more measurement points (Table S4). For this reason, as well as computational difficulties with applying GMM for categorical variables, it was decided to categorise the trajectories of dyspnoea manually, based on whether the participant most of the time did or did not exceed the cutoff score (= 'severe' trajectory), while accounting for missing observations.

This resulted in one class typified by no severity (*n* = 376, 75%) and one class typified by consistent severe dyspnoea (*n* = 123, 25%). See Figure S13 for an alluvial plot that visualises these trajectories.

Supplementary Table S4 Frequencies of dyspnoea severity

Number of measurements with mMRC scores ≥2	Participants (*N* = 500)
0	332
1	44
2	42
3	56
4	25
No measurements	1

Fig. S13 Alluvial plot showing the flow of dichotomised dyspnoea scores (severe/not severe)

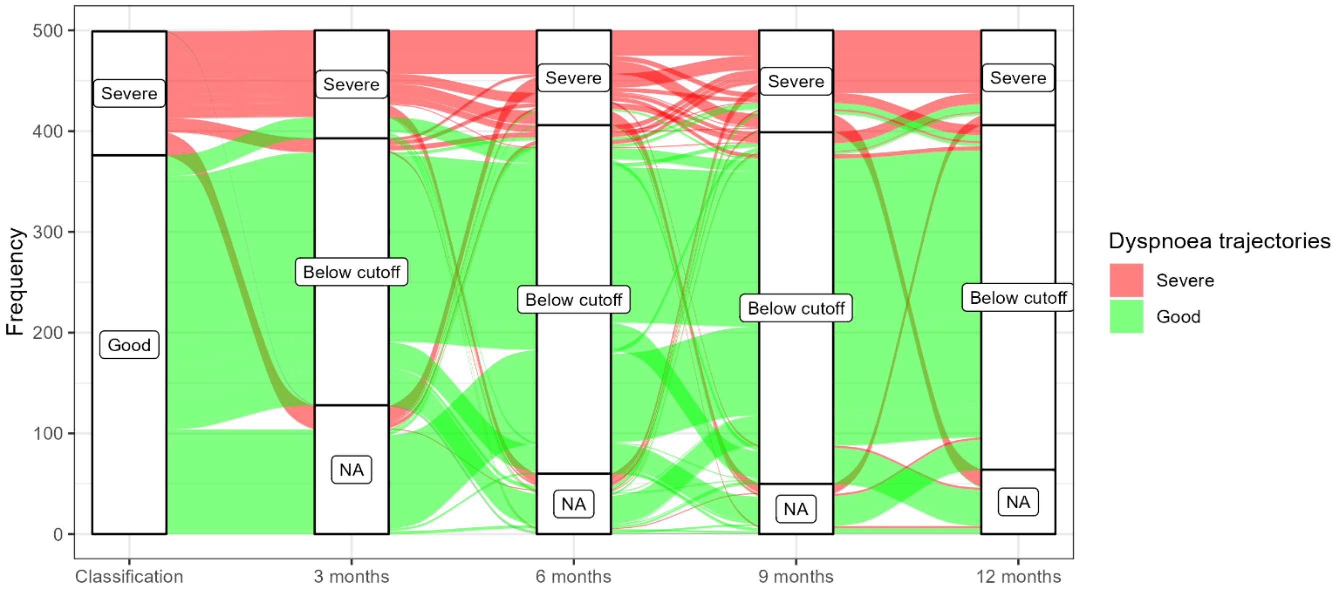

Note. The number of observations were: 372 (74.4% out of the possible 500) for 3 months, 440 (88%) for 6 months, 450 (90%) for 9 months, and 436 (87.2%) for 12 months. In total 1698 (84.9% out of 2000) observations were included.

Drop-out analysis

Supplementary Table S5 Characteristics of included and dropped-out participants

	Included sample (N = 500)	Drop-outs (N = 194)	p
Age in years, *M* (*SD*)	63.9 (12.4)	63.6 (12.4)	0.8
Sex, *n* (%)			0.9
Men	315 (63%)	121 (62%)	
Woman	185 (37%)	73 (38%)	
BMI, *M* (*SD*)	28.5 (4.5%)	28.6 (5.3%)	0.7
BMI class, *n* (%)			**0.043**
Underweight (<18.5)	0 (0%)	2 (1%)	
Normal weight (18.5 – 25)	99 (20%)	44 (23%)	
Overweight (25 – 30)	239 (49%)	78 (41%)	
Obesity (>30)	151 (31%)	68 (35%)	
Education, *n* (%)			0.6
Low	114 (23%)	52 (27%)	
Medium	232 (47%)	85 (44%)	
High	145 (30%)	57 (29%)	
Comorbidity, *n* (%)			0.051
0	112 (23%)	28 (14%)	
1	209 (42%)	93 (48%)	
2	97 (20%)	48 (25%)	
≥3	74 (15%)	25 (13%)	
PCS at 3 months, *M* (*SD*) [a]	37.5 (10.9)	37.7 (11.2)	>0.9
PCS at 12 months, *M* (*SD*) [b]	40.9 (11.8)	42.4 (11.7)	0.4
MCS at 3 months, *M* (*SD*) [a]	48.6 (10.7)	46.6 (10.5)	0.092
MCS at 12 months, *M* (*SD*) [b]	51.3 (10.3)	49.1 (9.7)	0.13
SFQ ≥18 at 3 months, *n* (%) [c]	215 (57%)	68 (64%)	0.2
SFQ ≥18 at 12 months, *n* (%) [d]	206 (45%)	25 (46%)	0.9
mMRC ≥2 at 3 months, *n* (%) [e]	107 (29%)	23 (23%)	0.2
mMRC ≥2 at 12 months, *n* (%) [f]	94 (22%)	11 (22%)	0.9

Note. Drop-out numbers based on participants with at least one observation, but with fewer than three observations in the included dataset. p-value for t-test or Pearson's χ^2 test for class differences. Missing values of characteristics are not indicated as they comprise a small proportion (<3%), except: [a] $n_{missing}$ = 126 & 95 for included and drop-outs respectively; [b] $n_{missing}$ = 48 & 144; [c] $n_{missing}$ = 121 & 88; [d] $n_{missing}$ = 45 & 140; [e] $n_{missing}$ = 128 & 92; [e] $n_{missing}$ = 64 & 145

Interpretation: Except for a minor, statistically significant difference for BMI classes, there were no differences between drop-outs and included participants. Drop-outs appear to have slightly fewer comorbidites and less overweight (yet more underweight), but overall do not appear to be more or less healthy. No differences in HRQoL or symptom severity could be noted (altough interpretation is troubled by substantial missing values).

Distributions of outcome measures

Supplementary Table S6 Distribution of observed outcomes by measurement points and trajectory

Trajectory	All measurements			3m			6m			9m			12m		
	n	***M***	***SD***	***n***	***M***	***SD***	***n***	***M***	***SD***	***n***	***M***	***SD***	***n***	***M***	***SD***
**Physical HRQoL (PCS)**															
Total sample (N = 500)	1716 (85.8%)	39.9	11.4	374 (74.8%)	37.5	10.9	446 (89.2%)	40.0	11.1	457 (94.4%)	40.6	11.6	452 (90.4%)	39.8	11.8
Stable good (*N* = 79)	-	-	-	56	53.5	3.4	72	54.8	2.9	72	54.9	3.4	74	55.1	2.6
Improving (*N* = 201)	-	-	-	152	39.9	7.8	181	43.2	7.7	187	45.1	7.7	184	47.8	5.2
Stable low (*N* = 220)	-	-	-	166	30.8	7.8	193	31.5	7.9	198	31.8	8.2	194	29.1	5.7
**Mental HRQoL (MCS)**															
Total sample (N = 500)	1716 (85.8%)	50.3	10.3	374 (74.8%)	48.6	10.7	446 (89.2%)	49.9	10.4	457 (94.4%)	50.9	10.0	452 (90.4%)	51.3	10.0
Stable good (N = 213)	-	-	-	156	57.8	4.1	188	58.2	3.9	200	57.9	4.4	196	58.7	4.0
Improving (N = 73)	-	-	-	56	42.6	4.5	65	52.5	5.2	70	54.4	4.1	65	52.9	4.5
Middle declining (N = 84)	-	-	-	70	51.5	5.2	75	45.9	7.4	74	43.3	7.6	73	47.7	6.5
Stable low (N = 130)	-	-	-	92	34.6	6.1	118	37.9	8.4	111	41.3	10.1	118	40.5	10.0
**Fatigue (SFQ)**															
Total sample (N = 500)	1734 (86.7%)	16.5	6.6	379 (75.8%)	17.8	6.3	450 (90%)	16.8	6.5	454 (90.8%)	16.0	6.8	451 (90.2%)	15.9	6.8
High-severe (N = 91)	-	-	-	71	25.2	1.9	81	24.9	2.3	77	24.5	1.9	81	24.5	2.2
Low-severe (N = 133)	-	-	-	105	21.1	2.7	123	20.3	2.4	120	20.4	2.5	110	20.4	2.5
Improving (N = 199)	-	-	-	146	15.7	4.2	178	14.4	4.1	183	13.4	4.6	189	13.2	4.2
No fatigue (N = 77)	-	-	-	57	7.9	2.0	68	6.8	2.3	71	6.2	2.1	71	6.5	2.0

Note. Percentages are the relative number of observations included of the total sample, i.e. out of the possible 2000 for all measurements and out of 500 for each follow-up measurement.

Trajectories of individuals per outcome and class

Fig. S14 Individual trajectories of physical HRQoL (PCS)

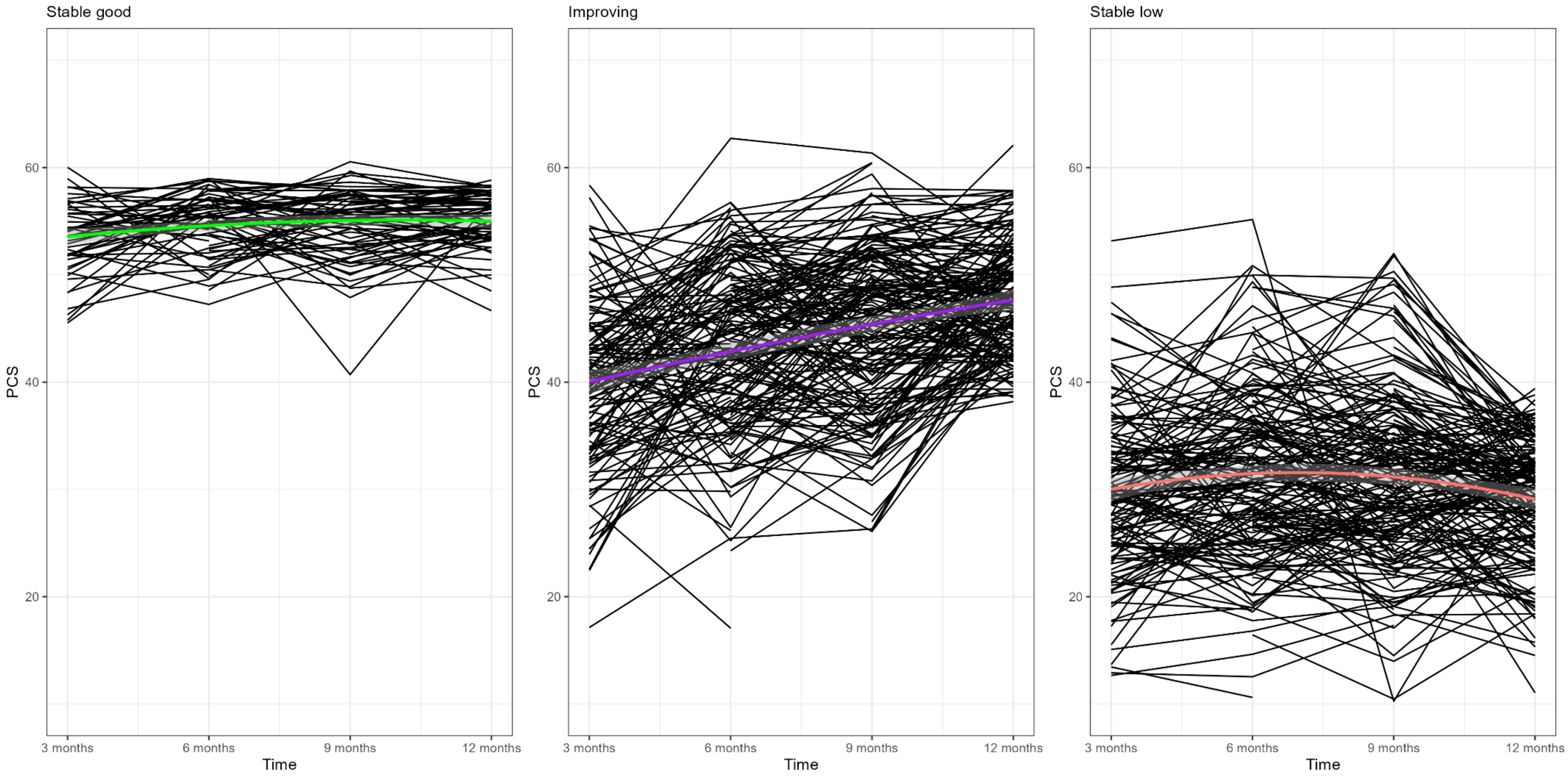

Fig. S15 Individual trajectories of mental HRQoL (MCS)

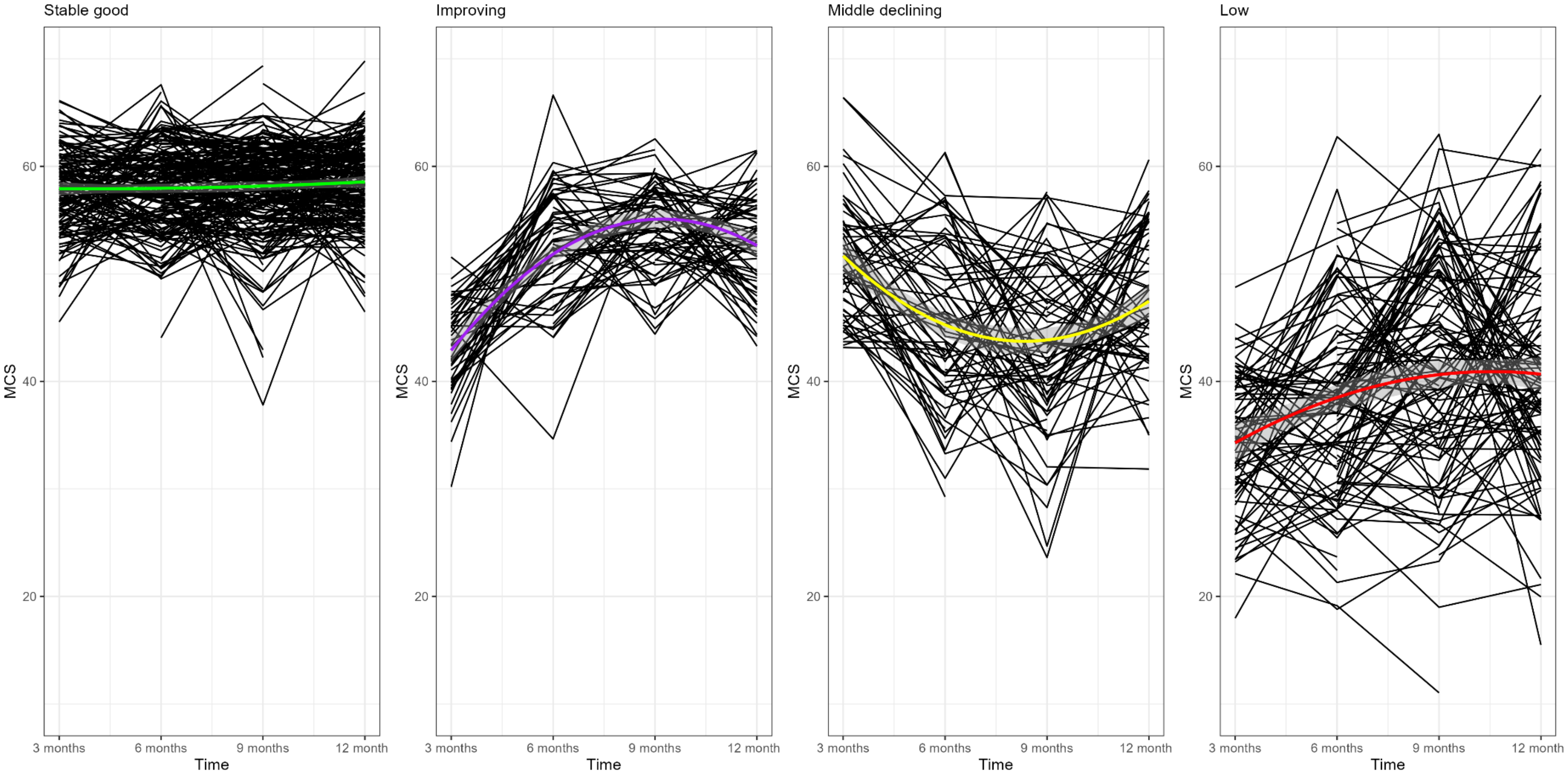

Fig. S16 Individual trajectories of fatigue (SFQ)

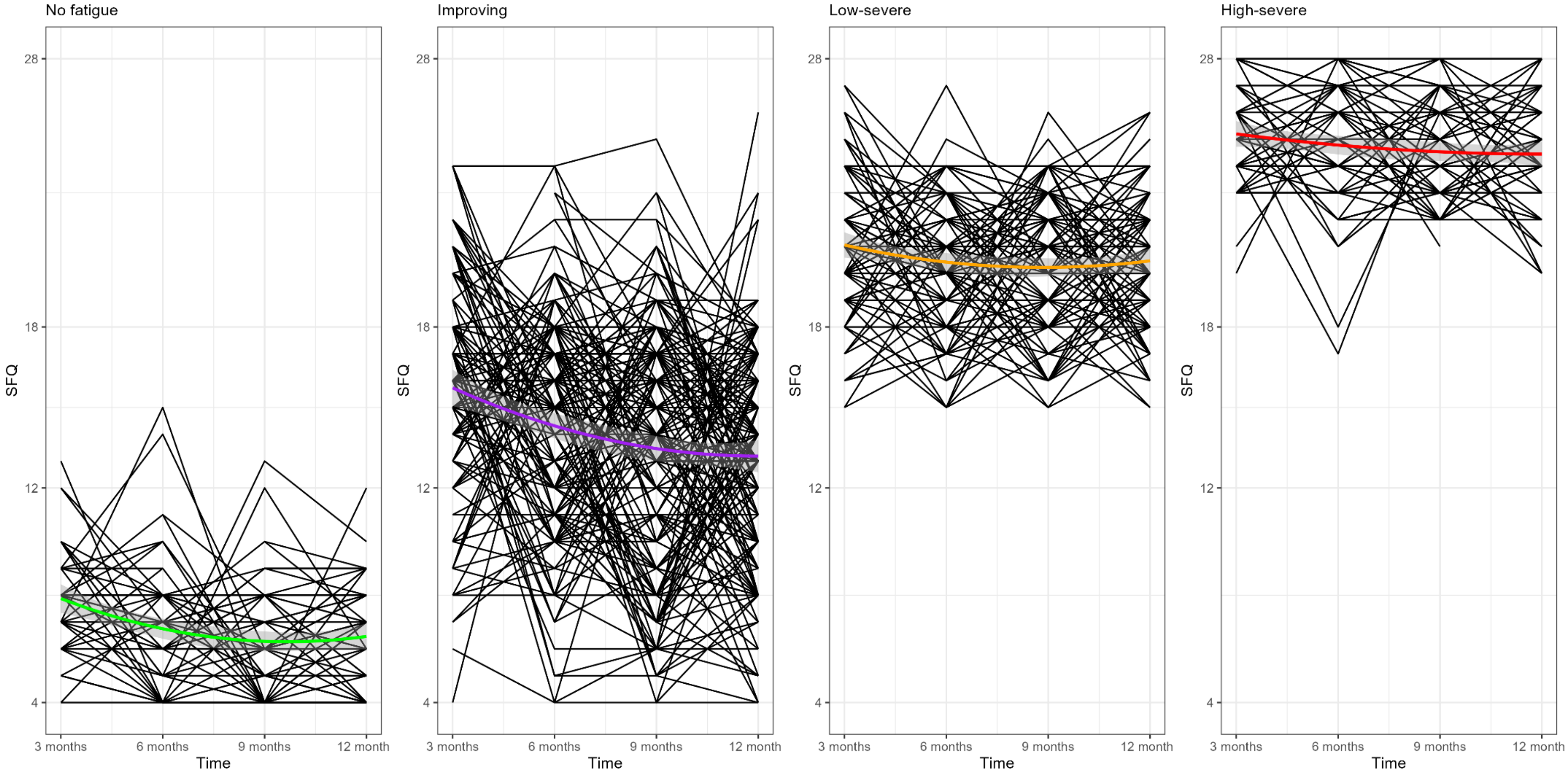

Characteristics per outcome trajectories

Supplementary Table S7 Characteristics of health-related quality of life trajectories

**Characteristic**	**Physical HRQoL**				**Mental HRQoL**				
	Stable good trajectory ($N = 79$)	Improving trajectory ($N = 201$)	Stable low trajectory ($N = 220$)	p	Stable good trajectory ($N = 213$)	Improving trajectory ($N = 73$)	Middle declining trajectory ($N = 84$)	Low trajectory ($N = 130$)	p
Age in years, *M* (*SD*)	62.4 (10.6)	61.4 (11.5)	66.7 (9.9)	<0.001	64.9 (10.6)	64.4 (11.2)	63.5 (10.0)	62.3 (11.7)	0.20
Sex, *n* (%)				0.01					0.30
Men	58 (73%)	135 (67%)	121 (55%)		138 (65%)	41 (57%)	59 (69%)	77 (59%)	
Woman	21 (27%)	66 (33%)	98 (45%)		75 (35%)	31 (43%)	26 (31%)	53 (41%)	
BMI class, *n* (%)				<0.001					0.30
Normal weight	27 (35%)	37 (19%)	35 (16%)		50 (24%)	12 (17%)	16 (19%)	21 (17%)	
Overweight	39 (50%)	112 (57%)	88 (41%)		104 (50%)	38 (54%)	36 (43%)	61 (48%)	
Obesity	12 (15%)	48 (24%)	91 (43%)		55 (26%)	20 (29%)	32 (38%)	44 (35%)	
Education, *n* (%)				<0.001					0.20
Low	15 (19%)	37 (19%)	62 (29%)		39 (19%)	20 (28%)	22 (26%)	33 (26%)	
Medium	26 (33%)	96 (49%)	10 (51%)		101 (48%)	28 (39%)	45 (54%)	58 (46%)	
High	37 (47%)	64 (32%)	44 (20%)		70 (33%)	23 (32%)	17 (20%)	36 (28%)	
Comorbidity, *n* (%)				<0.001					0.01
0	34 (44%)	55 (28%)	23 (10%)		59 (28%)	16 (23%)	11 (13%)	26 (20%)	
1	32 (41%)	98 (49%)	79 (37%)		93 (45%)	36 (51%)	38 (45%)	42 (33%)	
2	11 (14%)	26 (13%)	60 (28%)		36 (17%)	10 (14%)	19 (23%)	32 (25%)	
≥3	1 (1%)	19 (10%)	54 (25%)		22 (10%)	9 (13%)	16 (19%)	27 (22%)	

Notes. p-value for one-way analysis of variance (ANOVA) or Pearson's χ^2 test for class differences. Missing values of characteristics are not indicated as they comprise a small proportion (<3%) of the total sample ($N = 500$).

Supplementary Table S8 Characteristics of fatigue and dyspnoea trajectories

Characteristic	Fatigue					Dyspnoea		
	No fatigue trajectory ($N = 77$)	Improving trajectory ($N = 199$)	Low-severe trajectory ($N = 133$)	High-severe trajectory ($N = 91$)	*p*	No dyspnoea trajectory ($N = 376$)	Severe trajectory ($N = 123$)	*P*
Age in years, *M* (*SD*)	65.4 (10.3)	64.1 (10.7)	62.9 (11.9)	63.7 (10.4)	0.50	63.0 (10.9)	66.5 (10.7)	0.002
Sex, *n* (%)					0.40			0.04
Men	37 (48%)	101 (51%)	75 (56%)	53 (58%)		246 (65%)	68 (55%)	
Woman	40 (52%)	98 (49%)	58 (44%)	38 (42%)		130 (35%)	55 (45%)	
BMI class, *n* (%)					<0.001			<0.001
Normal weight	27 (36%)	34 (17%)	22 (17%)	16 (18%)		80 (22%)	19 (16%)	
Overweight	41 (54%)	111 (57%)	61 (47%)	26 (30%)		199 (54%)	39 (33%)	
Obesity	8 (11%)	51 (26%)	46 (36%)	46 (52%)		90 (24%)	61 (51%)	
Education, *n* (%)					<0.001			<0.001
Low	17 (22%)	40 (20%)	26 (20%)	31 (35%)		73 (20%)	40 (33%)	
Medium	24 (32%)	96 (49%)	76 (58%)	36 (41%)		169 (46%)	63 (53%)	
High	35 (46%)	60 (31%)	29 (22%)	21 (24%)		128 (35%)	17 (14%)	
Comorbidity, *n* (%)					<0.001			<0.001
0	26 (34%)	58 (30%)	17 (13%)	11 (12%)		99 (27%)	12 (10%)	
1	38 (50%)	91 (46%)	49 (37%)	31 (35%)		175 (47%)	34 (28%)	
2	9 (12%)	28 (14%)	36 (27%)	24 (27%)		62 (17%)	35 (29%)	
≥3	3 (3.9%)	19 (9.7%)	29 (22%)	23 (26%)		35 (9%)	39 (33%)	
Respiratory condition						62 (17%)	67 (56%)	<0.001

Notes. *p*-value for one-way analysis of variance (ANOVA) or Pearson's χ^2 test for class differences. Missing values of characteristics are not indicated as they comprise a small proportion (<3%) of the total sample ($N = 500$).

R syntax

Note: for the sake of conciseness, only the complete codes for all investigated models are described in full. For all other parts of the code (e.g. plot visualisation), see PCS for the example: the only differences are the outcome (PCS, MCS or SFQ).

```
# Used packages
library(haven)
library(tidyverse)
library(dplyr)
library(lcmm)
library(ggplot2)
library(gridExtra)

# Dataset
Data_long <- read_sav("PC_long.sav")
data <- v5_long %>%
  select(ID, Time, PCS, MCS, SFQ)

# set the seed for random number generator, so results can be reproduced:
set.seed(2023)
```

# LCGA

```
# Physical HRQoL
PCS1 <- hlme(PCS ~ Time, subject = "ID", ng = 1, data = data)
PCS2 <- gridsearch(rep = 100, maxiter = 10, minit = PCS1,
                   hlme(PCS ~ Time, subject = "ID", ng = 2, data = data,
                        mixture = ~ Time))
PCS3 <- gridsearch(rep = 100, maxiter = 10, minit = PCS1,
                   hlme(PCS ~ Time, subject = "ID", ng = 3, data = data,
                        mixture = ~ Time))
PCS4 <- gridsearch(rep = 100, maxiter = 10, minit = PCS1,
                   hlme(PCS ~ Time, subject = "ID", ng = 4, data = data,
                        mixture = ~ Time))
PCS5 <- gridsearch(rep = 100, maxiter = 10, minit = PCS1,
                   hlme(PCS ~ Time, subject = "ID", ng = 5, data = data,
                        mixture = ~ Time))

QPCS1 <- hlme(PCS ~ Time+I(Time^2), subject = "ID", ng = 1, data = data)
QPCS2 <- gridsearch(rep = 100, maxiter = 10, minit = QPCS1,
                    hlme(PCS ~ Time+I(Time^2), subject = "ID", ng = 2, data = data,
```

```
                    mixture = ~ Time+I(Time^2)))
QPCS3 <- gridsearch(rep = 100, maxiter = 10, minit = QPCS1,
                  hlme(PCS ~ Time+I(Time^2), subject = "ID", ng = 3, data = data,
                    mixture = ~ Time+I(Time^2)))
QPCS4 <- gridsearch(rep = 100, maxiter = 10, minit = QPCS1,
                  hlme(PCS ~ Time+I(Time^2), subject = "ID", ng = 4, data = data,
                    mixture = ~ Time+I(Time^2)))
QPCS5 <- gridsearch(rep = 100, maxiter = 10, minit = QPCS1,
                  hlme(PCS ~ Time+I(Time^2), subject = "ID", ng = 5, data = data,
                    mixture = ~ Time+I(Time^2)))

## Overview of indices for Physical HRQoL models
# Linear Physical HRQoL LCGA
summarytable(PCS1, PCS2, PCS3, PCS4, PCS5,
            which = c("G", "loglik", "conv", "npm", "AIC", "BIC", "SABIC",
                 "entropy","ICL", "%class"))
summaryplot(PCS1, PCS2, PCS3, PCS4, PCS5, which = c("BIC", "SABIC", "entropy"))

# Quadratic Physical HRQoL LCGA
summarytable(QPCS1, QPCS2, QPCS3, QPCS4, QPCS5,
            which = c("G", "loglik", "conv", "npm", "AIC", "BIC", "SABIC",
                 "entropy","ICL", "%class"))
summaryplot(QPCS1, QPCS2, QPCS3, QPCS4, QPCS5, which = c("BIC", "SABIC", "entropy"))

# posterior probabilities Physical HRQoL
postprob(PCS2)
postprob(PCS3)
postprob(PCS4)
postprob(PCS5)

postprob(QPCS2)
postprob(QPCS3)
postprob(QPCS4)
postprob(QPCS5)

## Plots of Physical HRQoL trajectories
### Linear
PCS_L1 <- data %>%
  select(ID, Time, PCS)
peoplePCS_L1 <- as.data.frame(PCS2$pprob[,1:2])
```

```
PCS_L1$group <- factor(peoplePCS_L1$class[sapply(as.numeric(PCS_L1$ID),
                                 function(x) which(peoplePCS_L1$ID == x))])
PCS_L2 <- data %>%
  select(ID, Time, PCS)
peoplePCS_L2 <- as.data.frame(PCS3$pprob[,1:2])
PCS_L2$group <- factor(peoplePCS_L2$class[sapply(as.numeric(PCS_L2$ID),
                                 function(x) which(peoplePCS_L2$ID == x))])
PCS_L3 <- data %>%
  select(ID, Time, PCS)
peoplePCS_L3 <- as.data.frame(PCS4$pprob[,1:2])
PCS_L3$group <- factor(peoplePCS_L3$class[sapply(as.numeric(PCS_L3$ID),
                                 function(x) which(peoplePCS_L3$ID == x))])
PCS_L4 <- data %>%
  select(ID, Time, PCS)
peoplePCS_L4 <- as.data.frame(PCS5$pprob[,1:2])
PCS_L4$group <- factor(peoplePCS_L4$class[sapply(as.numeric(PCS_L4$ID),
                                 function(x) which(peoplePCS_L4$ID == x))])

# plots for times from 1 to 4
pLP1 <- ggplot(PCS_L1, aes(Time, PCS, colour = group, linetype = group)) +
  geom_smooth(aes(group = group), method = "lm",
              size = 1, se = T) +
  scale_y_continuous(limits = c(0,100)) +
    scale_x_continuous(labels=c("1" = "3 months", "2" = "6 months",
                          "3" = "9 months", "4" = "12 months")) +
  labs(x = "Time", y = "PCS") +
  theme_bw() +
  theme(plot.title = element_text(size=11))

pLP2 <- ggplot(PCS_L2, aes(Time, PCS, colour = group, linetype = group)) +
  geom_smooth(aes(group = group), method = "lm",
              size = 1, se = T) +
  scale_y_continuous(limits = c(0,100)) +
    scale_x_continuous(labels=c("1" = "3 months", "2" = "6 months",
                          "3" = "9 months", "4" = "12 months")) +
  labs(x = "Time", y = "PCS") +
  theme_bw() +
  theme(plot.title = element_text(size=11))

pLP3 <- ggplot(PCS_L3, aes(Time, PCS, colour = group, linetype = group)) +
```

```
  geom_smooth(aes(group = group), method = "lm",
              size = 1, se = T) +
  scale_y_continuous(limits = c(0,100)) +
    scale_x_continuous(labels=c("1" = "3 months", "2" = "6 months",
                         "3" = "9 months", "4" = "12 months")) +
  labs(x = "Time", y = "PCS") +
  theme_bw() +
  theme(plot.title = element_text(size=11))

pLP4 <- ggplot(PCS_L4, aes(Time, PCS, colour = group, linetype = group)) +
  geom_smooth(aes(group = group), method = "lm",
              size = 1, se = T) +
  scale_y_continuous(limits = c(0,100)) +
    scale_x_continuous(labels=c("1" = "3 months", "2" = "6 months",
                         "3" = "9 months", "4" = "12 months")) +
  labs(x = "Time", y = "PCS") +
  theme_bw() +
  theme(plot.title = element_text(size=11))

grid.arrange(pLP1, pLP2, pLP3, pLP4, ncol = 2,
  top = "Observed Linear Trajectories of Latent Classes in Physical HRQoL (LCGA)")

### Quadratic
PCS_Q1 <- data %>%
  select(ID, Time, PCS)
peoplePCS_Q1 <- as.data.frame(QPCS2$pprob[,1:2])
PCS_Q1$group <- factor(peoplePCS_Q1$class[sapply(as.numeric(PCS_Q1$ID),
                           function(x) which(peoplePCS_Q1$ID == x))])
PCS_Q2 <- data %>%
  select(ID, Time, PCS)
peoplePCS_Q2 <- as.data.frame(QPCS3$pprob[,1:2])
PCS_Q2$group <- factor(peoplePCS_Q2$class[sapply(as.numeric(PCS_Q2$ID),
                           function(x) which(peoplePCS_Q2$ID == x))])
PCS_Q3 <- data %>%
  select(ID, Time, PCS)
peoplePCS_Q3 <- as.data.frame(QPCS4$pprob[,1:2])
PCS_Q3$group <- factor(peoplePCS_Q3$class[sapply(as.numeric(PCS_Q3$ID),
                           function(x) which(peoplePCS_Q3$ID == x))])
PCS_Q4 <- data %>%
  select(ID, Time, PCS)
```

```
peoplePCS_Q4 <- as.data.frame(QPCS5$pprob[,1:2])
PCS_Q4$group <- factor(peoplePCS_Q4$class[sapply(as.numeric(PCS_Q4$ID),
                                  function(x) which(peoplePCS_Q4$ID == x))])

# plots for times from 1 to 4
pQP1 <- ggplot(PCS_Q1, aes(Time, PCS, colour = group, linetype = group)) +
  geom_smooth(aes(group = group), method = "lm", formula = y ~ poly(x, 2),
            size = 1, se = T) +
  scale_y_continuous(limits = c(0,100)) +
    scale_x_continuous(labels=c("1" = "3 months", "2" = "6 months",
                         "3" = "9 months", "4" = "12 months")) +
  labs(x = "Time", y = "PCS") +
  theme_bw() +
  theme(plot.title = element_text(size=11))

pQP2 <- ggplot(PCS_Q2, aes(Time, PCS, colour = group, linetype = group)) +
  geom_smooth(aes(group = group), method = "lm", formula = y ~ poly(x, 2),
            size = 1, se = T) +
  scale_y_continuous(limits = c(0,100)) +
    scale_x_continuous(labels=c("1" = "3 months", "2" = "6 months",
                         "3" = "9 months", "4" = "12 months")) +
  labs(x = "Time", y = "PCS") +
  theme_bw() +
  theme(plot.title = element_text(size=11))

pQP3 <- ggplot(PCS_Q3, aes(Time, PCS, colour = group, linetype = group)) +
  geom_smooth(aes(group = group), method = "lm", formula = y ~ poly(x, 2),
            size = 1, se = T) +
  scale_y_continuous(limits = c(0,100)) +
    scale_x_continuous(labels=c("1" = "3 months", "2" = "6 months",
                         "3" = "9 months", "4" = "12 months")) +
  labs(x = "Time", y = "PCS") +
  theme_bw() +
  theme(plot.title = element_text(size=11))

pQP4 <- ggplot(PCS_Q4, aes(Time, PCS, colour = group, linetype = group)) +
  geom_smooth(aes(group = group), method = "lm", formula = y ~ poly(x, 2),
            size = 1, se = T) +
  scale_y_continuous(limits = c(0,100)) +
    scale_x_continuous(labels=c("1" = "3 months", "2" = "6 months",
```

```
                    "3" = "9 months", "4" = "12 months")) +
  labs(x = "Time", y = "PCS") +
  theme_bw() +
  theme(plot.title = element_text(size=11))

grid.arrange(pQP1, pQP2, pQP3, pQP4, ncol = 2,
  top = "Observed Quadratic Trajectories of Latent Classes in physical HRQoL (LCGA)")

# Mental HRQoL
MCS1 <- hlme(MCS ~ Time, subject = "ID", ng = 1, data = data)
MCS2 <- gridsearch(rep = 100, maxiter = 10, minit = MCS1,
          hlme(MCS ~ Time, subject = "ID", ng = 2, data = data,
             mixture = ~ Time))
MCS3 <- gridsearch(rep = 100, maxiter = 10, minit = MCS1,
          hlme(MCS ~ Time, subject = "ID", ng = 3, data = data,
             mixture = ~ Time))
MCS4 <- gridsearch(rep = 100, maxiter = 10, minit = MCS1,
          hlme(MCS ~ Time, subject = "ID", ng = 4, data = data,
             mixture = ~ Time))
MCS5 <- gridsearch(rep = 100, maxiter = 10, minit = MCS1,
          hlme(MCS ~ Time, subject = "ID", ng = 5, data = data,
             mixture = ~ Time))

QMCS1 <- hlme(MCS ~ Time+I(Time^2), subject = "ID", ng = 1, data = data)
QMCS2 <- gridsearch(rep = 100, maxiter = 10, minit = QMCS1,
          hlme(MCS ~ Time+I(Time^2), subject = "ID", ng = 2, data = data,
             mixture = ~ Time+I(Time^2)))
QMCS3 <- gridsearch(rep = 100, maxiter = 10, minit = QMCS1,
          hlme(MCS ~ Time+I(Time^2), subject = "ID", ng = 3, data = data,
             mixture = ~ Time+I(Time^2)))
QMCS4 <- gridsearch(rep = 100, maxiter = 10, minit = QMCS1,
          hlme(MCS ~ Time+I(Time^2), subject = "ID", ng = 4, data = data,
             mixture = ~ Time+I(Time^2)))
QMCS5 <- gridsearch(rep = 100, maxiter = 10, minit = QMCS1,
          hlme(MCS ~ Time+I(Time^2), subject = "ID", ng = 5, data = data,
             mixture = ~ Time+I(Time^2)))
QMCS5 <- gridsearch(rep = 100, maxiter = 10, minit = QMCS1,
          hlme(MCS ~ Time+I(Time^2), subject = "ID", ng = 5, data = data,
             mixture = ~ Time+I(Time^2)))
```

```
# Fatigue
SFQ1 <- hlme(SFQ ~ Time, subject = "ID", ng = 1, data = data)
SFQ2 <- gridsearch(rep = 100, maxiter = 10, minit = SFQ1,
            hlme(SFQ ~ Time, subject = "ID", ng = 2, data = data,
              mixture = ~ Time))
SFQ3 <- gridsearch(rep = 100, maxiter = 10, minit = SFQ1,
            hlme(SFQ ~ Time, subject = "ID", ng = 3, data = data,
              mixture = ~ Time))
SFQ4 <- gridsearch(rep = 100, maxiter = 10, minit = SFQ1,
            hlme(SFQ ~ Time, subject = "ID", ng = 4, data = data,
              mixture = ~ Time))
SFQ5 <- gridsearch(rep = 100, maxiter = 10, minit = SFQ1,
            hlme(SFQ ~ Time, subject = "ID", ng = 5, data = data,
              mixture = ~ Time))
SFQ6 <- gridsearch(rep = 100, maxiter = 10, minit = SFQ1,
            hlme(SFQ ~ Time, subject = "ID", ng = 6, data = data,
              mixture = ~ Time))
SFQ7 <- gridsearch(rep = 100, maxiter = 10, minit = SFQ1,
            hlme(SFQ ~ Time, subject = "ID", ng = 7, data = data,
              mixture = ~ Time))

QSFQ1 <- hlme(SFQ ~ Time+I(Time^2), subject = "ID", ng = 1, data = data)
QSFQ2 <- gridsearch(rep = 100, maxiter = 10, minit = QSFQ1,
            hlme(SFQ ~ Time+I(Time^2), subject = "ID", ng = 2, data = data,
              mixture = ~ Time+I(Time^2)))
QSFQ3 <- gridsearch(rep = 250, maxiter = 40, minit = QSFQ1,
            hlme(SFQ ~ Time+I(Time^2), subject = "ID", ng = 3, data = data,
              mixture = ~ Time+I(Time^2)))
QSFQ4 <- gridsearch(rep = 250, maxiter = 40, minit = QSFQ1,
            hlme(SFQ ~ Time+I(Time^2), subject = "ID", ng = 4, data = data,
              mixture = ~ Time+I(Time^2)))
QSFQ5 <- gridsearch(rep = 250, maxiter = 40, minit = QSFQ1,
            hlme(SFQ ~ Time+I(Time^2), subject = "ID", ng = 5, data = data,
              mixture = ~ Time+I(Time^2)))
```

# GMM

```
# Physical HRQoL
lp1 <- hlme(PCS ~ Time, subject = "ID", random = ~1 + Time, ng = 1,
          data = data)
lp2 <- gridsearch(rep = 100, maxiter = 10, minit = lp1,
```

```
                    hlme(PCS ~ Time, subject = "ID", random = ~1 + Time,
                        ng = 2, data = data, mixture = ~ Time, nwg=T))
lp3 <- gridsearch(rep = 500, maxiter = 40, minit = lp1,
                    hlme(PCS ~ Time, subject = "ID", random = ~1 + Time,
                        ng = 3, data = data, mixture = ~ Time, nwg=T))
lp4 <- gridsearch(rep = 500, maxiter = 40, minit = lp1,
                    hlme(PCS ~ Time, subject = "ID", random = ~1 + Time,
                        ng = 4, data = data, mixture = ~ Time, nwg=T))
lp5 <- gridsearch(rep = 500, maxiter = 40, minit = lp1,
                    hlme(PCS ~ Time, subject = "ID", random = ~1 + Time,
                        ng = 5, data = data, mixture = ~ Time, nwg=T))

qp1 <- hlme(PCS ~ Time+I(Time^2), subject = "ID", random = ~1 + Time+I(Time^2),
            ng = 1, data = data)
qp2 <- gridsearch(rep = 100, maxiter = 10, minit = qp1,
                  hlme(PCS ~ Time+I(Time^2), subject = "ID",
                      random = ~1 + Time+I(Time^2), ng = 2, data = data,
                      mixture = ~ Time+I(Time^2), nwg=T))
qp3 <- gridsearch(rep = 1000, maxiter = 40, minit = qp1,
                  hlme(PCS ~ Time+I(Time^2), subject = "ID",
                      random = ~1 + Time+I(Time^2), ng = 3, data = data,
                      mixture = ~ Time+I(Time^2), nwg=T))
qp4 <- gridsearch(rep = 1000, maxiter = 40, minit = qp1,
                  hlme(PCS ~ Time+I(Time^2), subject = "ID",
                      random = ~1 + Time+I(Time^2), ng = 4, data = data,
                      mixture = ~ Time+I(Time^2), nwg=T))
qp5 <- gridsearch(rep = 1000, maxiter = 40, minit = qp1,
                  hlme(PCS ~ Time+I(Time^2), subject = "ID",
                      random = ~1 + Time+I(Time^2), ng = 5, data = data,
                      mixture = ~ Time+I(Time^2), nwg=T))

## Overview of indices for Physical HRQoL models
# Linear Physical HRQoL GMM
summarytable(lp1, lp2, lp3, lp4, lp5,
            which = c("G", "loglik", "conv", "npm", "AIC", "BIC", "SABIC",
                "entropy","ICL", "%class"))
summaryplot(lp1, lp2, lp3, lp4, lp5, which = c("BIC", "SABIC", "entropy"))

# Quadratic Physical HRQoL GMM
summarytable(qp1, qp2, qp3, qp4, qp5,
```

```
            which = c("G", "loglik", "conv", "npm", "AIC", "BIC", "SABIC",
                      "entropy","ICL", "%class"))
summaryplot(qp1, qp2, qp3, qp4, qp5, which = c("BIC", "SABIC", "entropy"))

# posterior probabilities Physical HRQoL
postprob(lp2)
postprob(lp3)
postprob(lp4)
postprob(lp5)

postprob(qp2)
postprob(qp3)
postprob(qp4)
postprob(qp5)

## Plots of Physical HRQoL trajectories
### Linear
PCS_L1 <- data %>%
  select(ID, Time, PCS)
peoplePCS_L1 <- as.data.frame(lp2$pprob[,1:2])
PCS_L1$group <- factor(peoplePCS_L1$class[sapply(as.numeric(PCS_L1$ID),
                              function(x) which(peoplePCS_L1$ID == x))])
PCS_L2 <- data %>%
  select(ID, Time, PCS)
peoplePCS_L2 <- as.data.frame(lp3$pprob[,1:2])
PCS_L2$group <- factor(peoplePCS_L2$class[sapply(as.numeric(PCS_L2$ID),
                              function(x) which(peoplePCS_L2$ID == x))])
PCS_L3 <- data %>%
  select(ID, Time, PCS)
peoplePCS_L3 <- as.data.frame(lp4$pprob[,1:2])
PCS_L3$group <- factor(peoplePCS_L3$class[sapply(as.numeric(PCS_L3$ID),
                              function(x) which(peoplePCS_L3$ID == x))])
PCS_L4 <- data %>%
  select(ID, Time, PCS)
peoplePCS_L4 <- as.data.frame(lp5$pprob[,1:2])
PCS_L4$group <- factor(peoplePCS_L4$class[sapply(as.numeric(PCS_L4$ID),
                              function(x) which(peoplePCS_L4$ID == x))])

# plots for times from 1 to 4
pLP1 <- ggplot(PCS_L1, aes(Time, PCS, colour = group, linetype = group)) +
```

```
  geom_smooth(aes(group = group), method = "lm",
              size = 1, se = T) +
  scale_y_continuous(limits = c(0,100)) +
    scale_x_continuous(labels=c("1" = "3 months", "2" = "6 months",
                              "3" = "9 months", "4" = "12 months")) +
  labs(x = "Time", y = "PCS") +
  theme_bw() +
  theme(plot.title = element_text(size=11))

pLP2 <- ggplot(PCS_L2, aes(Time, PCS, colour = group, linetype = group)) +
  geom_smooth(aes(group = group), method = "lm",
              size = 1, se = T) +
  scale_y_continuous(limits = c(0,100)) +
    scale_x_continuous(labels=c("1" = "3 months", "2" = "6 months",
                              "3" = "9 months", "4" = "12 months")) +
  labs(x = "Time", y = "PCS") +
  theme_bw() +
  theme(plot.title = element_text(size=11))

pLP3 <- ggplot(PCS_L3, aes(Time, PCS, colour = group, linetype = group)) +
  geom_smooth(aes(group = group), method = "lm",
              size = 1, se = T) +
  scale_y_continuous(limits = c(0,100)) +
    scale_x_continuous(labels=c("1" = "3 months", "2" = "6 months",
                              "3" = "9 months", "4" = "12 months")) +
  labs(x = "Time", y = "PCS") +
  theme_bw() +
  theme(plot.title = element_text(size=11))

pLP4 <- ggplot(PCS_L4, aes(Time, PCS, colour = group, linetype = group)) +
  geom_smooth(aes(group = group), method = "lm",
              size = 1, se = T) +
  scale_y_continuous(limits = c(0,100)) +
    scale_x_continuous(labels=c("1" = "3 months", "2" = "6 months",
                              "3" = "9 months", "4" = "12 months")) +
  labs(x = "Time", y = "PCS") +
  theme_bw() +
  theme(plot.title = element_text(size=11))

grid.arrange(pLP1, pLP2, pLP3, pLP4, ncol = 2,
```

```
  top = "Observed Linear Trajectories of Latent Classes in Physical HRQoL")

### Quadratic
PCS_Q1 <- data %>%
  select(ID, Time, PCS)
peoplePCS_Q1 <- as.data.frame(qp2$pprob[,1:2])
PCS_Q1$group <- factor(peoplePCS_Q1$class[sapply(as.numeric(PCS_Q1$ID),
                                  function(x) which(peoplePCS_Q1$ID == x))])
PCS_Q2 <- data %>%
  select(ID, Time, PCS)
peoplePCS_Q2 <- as.data.frame(qp3$pprob[,1:2])
PCS_Q2$group <- factor(peoplePCS_Q2$class[sapply(as.numeric(PCS_Q2$ID),
                                  function(x) which(peoplePCS_Q2$ID == x))])
PCS_Q3 <- data %>%
  select(ID, Time, PCS)
peoplePCS_Q3 <- as.data.frame(qp4$pprob[,1:2])
PCS_Q3$group <- factor(peoplePCS_Q3$class[sapply(as.numeric(PCS_Q3$ID),
                                  function(x) which(peoplePCS_Q3$ID == x))])
PCS_Q4 <- data %>%
  select(ID, Time, PCS)
peoplePCS_Q4 <- as.data.frame(qp5$pprob[,1:2])
PCS_Q4$group <- factor(peoplePCS_Q4$class[sapply(as.numeric(PCS_Q4$ID),
                                  function(x) which(peoplePCS_Q4$ID == x))])

# plots for times from 1 to 4
pQP1 <- ggplot(PCS_Q1, aes(Time, PCS, colour = group, linetype = group)) +
  geom_smooth(aes(group = group), method = "lm", formula = y ~ poly(x, 2),
            size = 1, se = T) +
  scale_y_continuous(limits = c(0,100)) +
    scale_x_continuous(labels=c("1" = "3 months", "2" = "6 months",
                          "3" = "9 months", "4" = "12 months")) +
  labs(x = "Time", y = "PCS") +
  theme_bw() +
  theme(plot.title = element_text(size=11))
```

```
pQP2 <- ggplot(PCS_Q2, aes(Time, PCS, colour = group, linetype = group)) +
  geom_smooth(aes(group = group), method = "lm", formula = y ~ poly(x, 2),
            size = 1, se = T) +
  scale_y_continuous(limits = c(0,100)) +
    scale_x_continuous(labels=c("1" = "3 months", "2" = "6 months",
                        "3" = "9 months", "4" = "12 months")) +
  labs(x = "Time", y = "PCS") +
  theme_bw() +
  theme(plot.title = element_text(size=11))

pQP3 <- ggplot(PCS_Q3, aes(Time, PCS, colour = group, linetype = group)) +
  geom_smooth(aes(group = group), method = "lm", formula = y ~ poly(x, 2),
            size = 1, se = T) +
  scale_y_continuous(limits = c(0,100)) +
    scale_x_continuous(labels=c("1" = "3 months", "2" = "6 months",
                        "3" = "9 months", "4" = "12 months")) +
  labs(x = "Time", y = "PCS") +
  theme_bw() +
  theme(plot.title = element_text(size=11))

pQP4 <- ggplot(PCS_Q4, aes(Time, PCS, colour = group, linetype = group)) +
  geom_smooth(aes(group = group), method = "lm", formula = y ~ poly(x, 2),
            size = 1, se = T) +
  scale_y_continuous(limits = c(0,100)) +
    scale_x_continuous(labels=c("1" = "3 months", "2" = "6 months",
                        "3" = "9 months", "4" = "12 months")) +
  labs(x = "Time", y = "PCS") +
  theme_bw() +
  theme(plot.title = element_text(size=11))

grid.arrange(pQP1, pQP2, pQP3, pQP4, ncol = 2,
  top = "Observed Quadratic Trajectories of Latent Classes in physical HRQoL")

# Mental HRQoL
lm1 <- hlme(MCS ~ Time, subject = "ID", random = ~1 + Time, ng = 1,
             data = data)
lm2 <- gridsearch(rep = 100, maxiter = 10, minit = lm1,
                  hlme(MCS ~ Time, subject = "ID", random = ~1 + Time,
                    ng = 2, data = data, mixture = ~ Time, nwg=T))
lm3 <- gridsearch(rep = 500, maxiter = 40, minit = lm1,
```

```
                  hlme(MCS ~ Time, subject = "ID", random = ~1 + Time,
                     ng = 3, data = data, mixture = ~ Time, nwg=T))
lm4 <- gridsearch(rep = 500, maxiter = 40, minit = lm1,
                  hlme(MCS ~ Time, subject = "ID", random = ~1 + Time,
                     ng = 4, data = data, mixture = ~ Time, nwg=T))
lm5 <- gridsearch(rep = 500, maxiter = 40, minit = lm1,
                  hlme(MCS ~ Time, subject = "ID", random = ~1 + Time,
                     ng = 5, data = data, mixture = ~ Time, nwg=T))

qm1 <- hlme(MCS ~ Time+I(Time^2), subject = "ID", random = ~1 + Time+I(Time^2),
            ng = 1, data = data)
qm2 <- gridsearch(rep = 100, maxiter = 10, minit = qm1,
                hlme(MCS ~ Time+I(Time^2), subject = "ID",
                    random = ~1 + Time+I(Time^2), ng = 2, data = data,
                    mixture = ~ Time+I(Time^2), nwg=T))
qm3 <- gridsearch(rep = 1000, maxiter = 40, minit = qm1,
                hlme(MCS ~ Time+I(Time^2), subject = "ID",
                    random = ~1 + Time+I(Time^2), ng = 3, data = data,
                    mixture = ~ Time+I(Time^2), nwg=T))
qm4 <- gridsearch(rep = 1000, maxiter = 40, minit = qm1,
                hlme(MCS ~ Time+I(Time^2), subject = "ID",
                    random = ~1 + Time+I(Time^2), ng = 4, data = data,
                    mixture = ~ Time+I(Time^2), nwg=T))
qm5 <- gridsearch(rep = 1000, maxiter = 40, minit = qm1,
                hlme(MCS ~ Time+I(Time^2), subject = "ID",
                    random = ~1 + Time+I(Time^2), ng = 5, data = data,
                    mixture = ~ Time+I(Time^2), nwg=T))

# Fatigue
lf1 <- hlme(SFQ ~ Time, subject = "ID", random = ~1 + Time, ng = 1,
            data = data)
lf2 <- gridsearch(rep = 100, maxiter = 10, minit = lf1,
                  hlme(SFQ ~ Time, subject = "ID", random = ~1 + Time,
                     ng = 2, data = data, mixture = ~ Time, nwg=T))
lf3 <- gridsearch(rep = 500, maxiter = 40, minit = lf1,
                  hlme(SFQ ~ Time, subject = "ID", random = ~1 + Time,
                     ng = 3, data = data, mixture = ~ Time, nwg=T))
lf4 <- gridsearch(rep = 500, maxiter = 40, minit = lf1,
                  hlme(SFQ ~ Time, subject = "ID", random = ~1 + Time,
                     ng = 4, data = data, mixture = ~ Time, nwg=T))
```

```
lf5 <- gridsearch(rep = 500, maxiter = 40, minit = lf1,
            hlme(SFQ ~ Time, subject = "ID", random = ~1 + Time,
              ng = 5, data = data, mixture = ~ Time, nwg=T))

qf1 <- hlme(SFQ ~ Time+I(Time^2), subject = "ID", random = ~1 + Time+I(Time^2),
       ng = 1, data = data)
qf2 <- gridsearch(rep = 100, maxiter = 10, minit = qf1,
           hlme(SFQ ~ Time+I(Time^2), subject = "ID",
             random = ~1 + Time+I(Time^2), ng = 2, data = data,
             mixture = ~ Time+I(Time^2), nwg=T))
qf3 <- gridsearch(rep = 1000, maxiter = 40, minit = qf1,
           hlme(SFQ ~ Time+I(Time^2), subject = "ID",
             random = ~1 + Time+I(Time^2), ng = 3, data = data,
             mixture = ~ Time+I(Time^2), nwg=T))
qf4 <- gridsearch(rep = 1000, maxiter = 40, minit = qf1,
           hlme(SFQ ~ Time+I(Time^2), subject = "ID",
             random = ~1 + Time+I(Time^2), ng = 4, data = data,
             mixture = ~ Time+I(Time^2), nwg=T))
qf5 <- gridsearch(rep = 1000, maxiter = 40, minit = qf1,
           hlme(SFQ ~ Time+I(Time^2), subject = "ID",
             random = ~1 + Time+I(Time^2), ng = 5, data = data,
             mixture = ~ Time+I(Time^2), nwg=T))
qf6 <- gridsearch(rep = 1000, maxiter = 40, minit = qf1,
           hlme(SFQ ~ Time+I(Time^2), subject = "ID",
             random = ~1 + Time+I(Time^2), ng = 6, data = data,
             mixture = ~ Time+I(Time^2), nwg=T))
```

GRoLTS Checklist

	Checklist item	Reported? (Page No.)
1.	Is the metric of time used in the statistical model reported?	Yes (2)
2.	Is information presented about the mean and variance of time within a wave?	Yes (2)
3a.	Is the missing data mechanism reported?	Yes (3)
3b.	Is a description provided of what variables are related to attrition/missing data?	Yes (2; 13; SM13)
3c.	Is a description provided of how missing data in the analyses were dealt with?	Yes (3)
4.	Is information about the distribution of the observed variables included?	Yes (SM14)
5.	Is the software mentioned?	Yes (3)
6a.	Are alternative specifications of within-class heterogeneity considered (e.g., LGCA vs. LGMM) and clearly documented? If not, was sufficient justification provided as to eliminate certain specifications from consideration?	Yes (SM2)
6b.	Are alternative specifications of the between-class differences in variance–covariance matrix structure considered and clearly documented? If not, was sufficient justification provided as to eliminate certain specifications from consideration?	Yes (SM2)
7.	Are alternative shape/functional forms of the trajectories described?	Yes (SM2)
8.	If covariates have been used, can analyses still be replicated?	Yes (3; 13)
9.	Is information reported about the number of random start values and final iterations included?	Yes (SM3-9; SM20-33)
10.	Are the model comparison (and selection) tools described from a statistical perspective?	Yes (SM2)
11.	Are the total number of fitted models reported, including a one-class solution?	Yes (SM3-9)
12.	Are the number of cases per class reported for each model (absolute sample size, or proportion)?	Yes (SM3-9)
13.	If classification of cases in a trajectory is the goal, is entropy reported?	Yes (SM3-9)
14a.	Is a plot included with the estimated mean trajectories of the final solution?	Yes (6)
14b.	Are plots included with the estimated mean trajectories for each model?	Yes (SM4-11)
14c.	Is a plot included of the combination of estimated means of the final model and the observed individual trajectories split out for each latent class?	Yes (SM15-17)
15.	Are characteristics of the final class solution numerically described (i.e., means, SD/SE, n, CI, etc.)?	Yes (SM18-19)
16.	Are the syntax files available (either in the appendix, supplementary materials, or from the authors)?	Yes (SM20-33)

Note. LGCA = latent class growth analysis; LGMM = latent growth mixture modelling; SM = Supplementary Material

STROBE Checklist

STROBE Statement—Checklist of items that should be included in reports of cohort studies

	Item No.	Recommendation	Page No.
**Title and abstract**	1	(a) Indicate the study's design with a commonly used term in the title or the abstract	Abstract
		(b) Provide in the abstract an informative and balanced summary of what was done and what was found	Abstract
**Introduction**			
Background/rationale	2	Explain the scientific background and rationale for the investigation being reported	1
Objectives	3	State specific objectives, including any prespecified hypotheses	1
**Methods**			
Study design	4	Present key elements of study design early in the paper	2
Setting	5	Describe the setting, locations, and relevant dates, including periods of recruitment, exposure, follow-up, and data collection	2
Participants	6	(a) Give the eligibility criteria, and the sources and methods of selection of participants. Describe methods of follow-up	2
		(b) For matched studies, give matching criteria and number of exposed and unexposed	N/A
Variables	7	Clearly define all outcomes, exposures, predictors, potential confounders, and effect modifiers. Give diagnostic criteria, if applicable	2-3
Data sources/ measurement	8*	For each variable of interest, give sources of data and details of methods of assessment (measurement). Describe comparability of assessment methods if there is more than one group	2; 4
Bias	9	Describe any efforts to address potential sources of bias	2
Study size	10	Explain how the study size was arrived at	2
Quantitative variables	11	Explain how quantitative variables were handled in the analyses. If applicable, describe which groupings were chosen and why	3
Statistical methods	12	(*a*) Describe all statistical methods, including those used to control for confounding	3
		(*b*) Describe any methods used to examine subgroups and interactions	3
		(*c*) Explain how missing data were addressed	3
		(*d*) If applicable, explain how loss to follow-up was addressed	3
		(*e*) Describe any sensitivity analyses	N/A
**Results**			
Participants	13	(a) Report numbers of individuals at each stage of study—eg numbers potentially eligible, examined for eligibility, confirmed eligible, included in the study, completing follow-up, and analysed	2
		(b) Give reasons for non-participation at each stage	2
		(c) Consider use of a flow diagram	N/A
Descriptive data	14	(a) Give characteristics of study participants (eg demographic, clinical, social) and information on exposures and potential confounders	5

		(b) Indicate number of participants with missing data for each variable of interest	3; SM12; SM14
		(c) Summarise follow-up time (eg, average and total amount)	2
Outcome Data	15*	Report numbers of outcome events or summary measures over time	5-7; SM14
Main Results	16	(*a*) Give unadjusted estimates and, if applicable, confounder-adjusted estimates and their precision (eg, 95% confidence interval). Make clear which confounders were adjusted for and why they were included	8; 10
		(*b*) Report category boundaries when continuous variables were categorized	2
		(*c*) If relevant, consider translating estimates of relative risk into absolute risk for a meaningful time period	N/A
Other analyses	17	Report other analyses done—eg analyses of subgroups and interactions, and sensitivity analyses	SM13; SM18-19
**Discussion**			
Key results	18	Summarise key results with reference to study objectives	10-11
Limitations	19	Discuss limitations of the study, taking into account sources of potential bias or imprecision. Discuss both direction and magnitude of any potential bias	13-14
Interpretation	20	Give a cautious overall interpretation of results considering objectives, limitations, multiplicity of analyses, results from similar studies, and other relevant evidence	11-13
Generalisability	21	Discuss the generalisability (external validity) of the study results	11-13
**Other Information**			
Funding	22	Give the source of funding and the role of the funders for the present study and, if applicable, for the original study on which the present article is based	18

*Give information separately for exposed and unexposed groups.
